# HSI-II Gene Cluster of *Pseudomonas syringae* pv. tomato DC3000 Encodes a Functional Type VI Secretion System Required for Interbacterial Competition

**DOI:** 10.3389/fmicb.2020.01118

**Published:** 2020-06-03

**Authors:** Ching-Fang Chien, Cheng-Ying Liu, Yew-Yee Lu, You-Hsing Sung, Kuo-Yau Chen, Nai-Chun Lin

**Affiliations:** Department of Agricultural Chemistry, National Taiwan University, Taipei, Taiwan

**Keywords:** *Pseudomonas syringe*, tomato speck disease, type VI secretion system, interbacterial competition, Hcp2

## Abstract

The type VI secretion system (T6SS) is a widespread bacterial nanoweapon used for delivery of toxic proteins into cell targets and contributes to virulence, anti-inflammatory processes, and interbacterial competition. In the model phytopathogenic bacterium *Pseudomonas syringae* pv. tomato (*Pst*) DC3000, two T6SS gene clusters, HSI-I and HSI-II, were identified, but their functions remain unclear. We previously reported that *hcp2*, located in HSI-II, is involved in competition with enterobacteria and yeast. Here, we demonstrated that interbacterial competition of *Pst* DC3000 against several Gram-negative plant-associated bacteria requires mainly HSI-II activity. By means of a systematic approach using in-frame deletion mutants for each gene in the HSI-II cluster, we identified genes indispensable for Hcp2 expression, Hcp2 secretion and interbacterial competition ability. Deletion of PSPTO_5413 only affected growth in interbacterial competition assays but not Hcp2 secretion, which suggests that PSPTO_5413 might be a putative effector. Moreover, PSPTO_5424, encoding a putative σ^54^-dependent transcriptional regulator, positively regulated the expression of all three operons in HSI-II. Our discovery that the HSI-II gene cluster gives *Pst* DC3000 the ability to compete with other plant-associated bacteria could help in understanding a possible mechanism of how phytopathogenic bacteria maintain their ecological niches.

## Introduction

Natural habitats do not always provide suitable conditions for microorganisms to survive; therefore, bacteria have evolved various secretion systems to adapt to changing environments by translocating effector proteins or DNA into the surrounding milieu or target cells (Costa et al., 2015). The type VI secretion system (T6SS) has been found in more than 25% of sequenced Gram-negative bacteria, and some strains even harbor multiple copies of T6SS-associated genes, which suggests its conserved but specialized roles in bacterial survival (Bingle et al., 2008; Coulthurst, 2019). Genes coding for core components of T6SS are usually clustered together and are considered acquired via horizontal gene transfer (Sarris et al., 2010; Borgeaud et al., 2015; Salomon et al., 2015; Thomas et al., 2017). Functional and structural analyses indicated that T6SS could assemble into a tubular structure similar to a contractile phage tail, in which an Hcp inner tube capped by VgrG/PAAR spike proteins and wrapped by a VipA/B (also known as HsiB/C, or TssB/C) sheath is polymerized on a membrane-associated baseplate complex (Lossi et al., 2013). A conformational change in the baseplate complex stimulates sheath contraction, thus leading to propelling of an Hcp/VgrG/PAAR puncturing device through cell membranes for effector secretion (Basler et al., 2012; Shneider et al., 2013; Zoued et al., 2016). Upon firing, the contracted VipA/B sheath is disassembled by a specialized AAA + ATPase ClpV to recycle VipA/B subunits for assembly of a new T6SS apparatus. The phage tail-like structure is stabilized by a membrane core complex containing TssL, M, and J-like proteins, and this complex could also function as a channel for inner tube passage after sheath contraction (Brunet et al., 2015). To quickly and efficiently respond to the environmental stimuli, T6SS in a subset of bacterial species can be regulated by threonine phosphorylation and posttranslational regulation via serine/threonine kinase PpkA, phosphatase PphA, and a forkhead-associated (FHA) domain-containing protein, Fha (Mougous et al., 2007; Lin et al., 2014).

After the term T6SS was coined in 2006 (Mougous et al., 2006; Pukatzki et al., 2006), the requirement of T6SS for virulence against eukaryotic hosts was described in diverse pathogens (Coulthurst, 2019). However, the contribution of T6SS to interbacterial competitiveness toward various bacterial species via delivery of toxic proteins into target cells highlights its pivotal role in growth fitness in the natural environment containing diverse microbiota (Hood et al., 2010; Hachani et al., 2016; Bernal et al., 2017; Allsopp et al., 2019). T6SS is also involved in limiting colonization and intestinal inflammation to maintain a non-pathogenic, symbiotic relationship of a pathobiont in the gastrointestinal tract within the host (Chow and Mazmanian, 2010), secretion of unknown proteins that impair formation of effective nodules on pea and vetch in *Rhizobium leguminosarum* (Bladergroen et al., 2003), fostering competence-mediated horizontal gene transfer in *Vibrio cholerae* (Blokesch, 2015; Borgeaud et al., 2015) and anti-fungal activities (Haapalainen et al., 2012; Marchi et al., 2013; Trunk et al., 2018). In addition to the contact-dependent T6SS activities described above, some T6SSs could function to deliver effectors into the extracellular environments for uptake of particular metal ions such as Zn^2+^ (Wang et al., 2015; Si et al., 2017), Mn^2+^ (Si et al., 2017), Fe^2+^ (Lin et al., 2017), and Cu^2+^ (Han et al., 2019), which further expands the functions of this versatile and multipurpose nanoweapon.

In addition to the core components involved in apparatus assembly and activation, T6SS functions also rely on secreted proteins, termed T6SS effectors. Secretome, bioinformatics and genetic analyses revealed T6SS effectors such as anti-eukaryotic or anti-bacterial toxins and extracellular metallophores. According to the mode of delivery, T6SS effectors can be divided into cargo and specialized effectors: the former non-covalently associate with specific Hcp, VgrG or a PAAR-containing protein, and the latter contain an effector domain covalently fused to the C-terminal domain of Hcp, VgrG or a PAAR protein (Durand et al., 2014). Binding to the narrow lumen of the inner tube (40Å in diameter) formed by stacked hexameric Hcp rings, Hcp-mediated cargo effectors are usually small (<25 kDa) (Silverman et al., 2013). In certain scenarios, chaperones or adaptors (such as proteins containing DUF1795, DUF2169, or DUF4123 domain) are also required for effector recruitment and secretion by T6SS (Coulthurst, 2019). Genes coding for cargo effectors and cognate chaperones/adaptors are likely to be found in the vicinity of *hcp*, *vgrG*, or *paar* genes (Durand et al., 2014; Ma et al., 2018; Coulthurst, 2019).

*Pseudomonas syringae* pathovar tomato (*Pst*) DC3000, a causative agent of tomato speck disease, has become one of the well-known model organisms to study plant–microbe interactions. The pathogenicity and virulence of *Pst* DC3000 depend on a functional type III secretion system, which delivers effectors into host cells to block the plant defense system or interfere with normal metabolism *in planta* (Xin and He, 2013). *In silico* analysis of genomes from six pathovars of *P. syringae* revealed that *Pst* DC3000 as well as *Pst* T1, *P. s.* pv. *tabaci* (*Psta*) ATCC 11528, and *P. s.* pv. *oryzae* 1–6 contain two putative T6SS clusters, Hcp secretion island 1 (HSI-I) and HSI-II. The expression of an *icmF* (*tssM*) ortholog in HSI-II of *Pst* DC3000 has been demonstrated (Sarris et al., 2010; Barret et al., 2011). Secretome analysis revealed that Hcp2 encoded in the HSI-II cluster was secreted in an *icmF*-dependent manner and plays a role in competition ability against enterobacteria and yeast. However, Hcp2 is not involved in *Pst* DC3000 virulence on tomato and Arabidopsis (Haapalainen et al., 2012). Nevertheless, the biological role of T6SS in phytopathogenic *Pst* DC3000 remains largely unknown and needs further investigation.

Dissection of T6SS function by using systematical mutagenesis of each gene in the T6SS cluster has been successfully applied in *Agrobacterium tumefaciens*, *Edwardsiella tarda*, and *V. cholerae* (Zheng and Leung, 2007; Zheng et al., 2011; Lin et al., 2013). In this study, to gain a broad view of T6SS activity in *Pst* DC3000, we first investigated whether HSI-I and HSI-II are both functional in interbacterial competition ability. After redefining the content of the HSI-II cluster, we analyzed mutated genes in this gene cluster. Detection of the hallmark T6SS-secreted protein Hcp2 combined with interbacterial competition assay allowed us to confirm core components and identify a regulator and a putative effector encoded in the HSI-II gene cluster. Our data shed light on understanding the role of T6SS in *Pst* DC3000 and also provide tools for further dissecting T6SS functions in *Pst* DC3000.

## Materials and Methods

### Bacterial Strains, Plasmids and Culture Media

Bacterial strains and plasmids used in this study are in Supplementary Table S1. *Pst* DC3000 strains were cultured in King’s B medium (KBM) supplemented with appropriate antibiotics or in *hrp*-inducing minimal media (HMM) substituted with different carbon sources at 28°C. *E. coli* strains were grown in Luria-Bertani (LB) medium at 37°C. The concentrations of antibiotics used were for gentamicin, 10 μg/mL; kanamycin, 50 μg/mL; rifampicin, 50 μg/mL; and tetracycline, 30 μg/mL.

### Construction of Mutations in *Pst* DC3000

The upstream and downstream sequences of each gene to be deleted were PCR-amplified with the primers listed in Supplementary Table S2. Crossover PCR was then performed to obtain a DNA fragment containing the upstream and downstream sequences for each gene and cloned into the suicide vector pK18*mobsac* (Schafer et al., 1994). The successful clones were then used to select deletion mutants in a SacB-based procedure as described (Haapalainen et al., 2012). From annotation information provided by the *Pseudomonas* Genome Database^1^, ΔHSI-I and ΔHSI-II were constructed by deleting PSPTO_2542 to 2554 and PSPTO_5414 to 5427, respectively.

### Construction of Plasmids for GUS Reporter Assay

Two and three promoters were predicted in HSI-I and HSI-II gene clusters, respectively (Supplementary Figure S1). The 500 bp fragments upstream of PSPTO_2542, PSPTO_2539, PSPTO_5427, PSPTO_5434, and PSPTO_5435 amplified with the primers listed in Supplementary Table S2 were cloned into pCHUB78, which contains a promoterless *uidA* downstream of a multiple cloning site, to obtain p*HSI-Ip* (P*HSI-I*:*uidA*), p*hcp1p* (P*hcp1*:*uidA*), p*HSI-II-ip* (P*HSI-II-i*:*uidA*), p*HSI-II-iip* (P*HSI-II-ii*:*uidA*), and p*HSI-II*-*hcp2p* (P*hcp2:uidA*). The resulting plasmids were then introduced into *Pst* DC3000, which was then grown in KBM from OD_600_ = 0.1 to 0.8 before cells were collected for 4-methylumbelliferyl-β-D-glucuronide (4-MUG)-based β-glucuronidase (GUS) reporter assay (Gallagher, 1992). The relative GUS activity is represented as a ratio of GUS activity obtained from the tested strain and the corresponding control strain carrying an empty vector.

### Interbacterial Competition Assay

**FIGURE 1:**
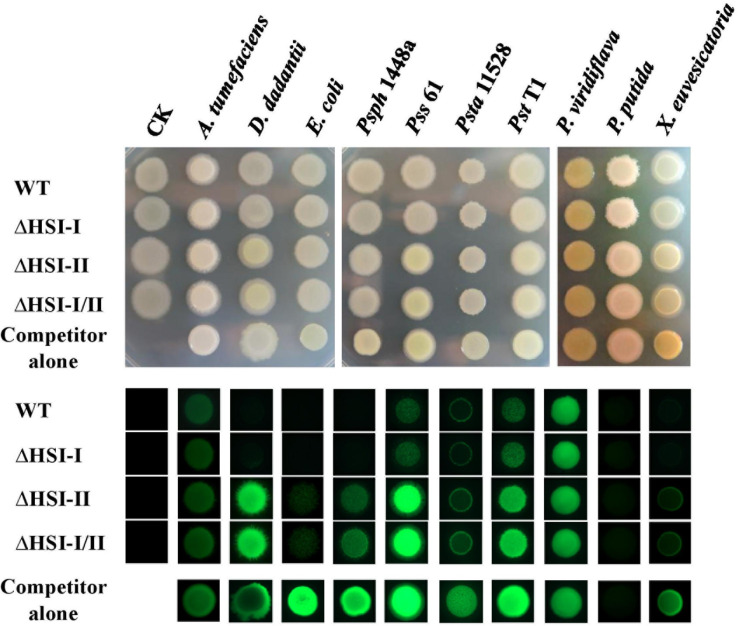
The HSI-II gene cluster is involved in the interbacterial competition ability of *Pst* DC3000. GFP-expressing competitor bacterial strains, including *Agrobacterium tumefaciens* C58, *Dickeya dadantii* CAS9, *E. coli* MG1655, *P. savastanoi* pv. phaseolicola (*Psph*) 1448a, *P. syringae* pv. syringae (*Psy*) 61, *P. s.* pv. tabaci (*Psta*) 11528, *Pst* T1, *P. viridiflava*, *P. putida* A7, and *X. euvescatoria* XvT147 were mixed with *Pst* DC3000 wild type (WT) or its derivative mutants in a 1:1 ratio. A 5 μL amount of the mixed bacterial solution was dropped onto KBM plates, then incubated at 28°C. Colony morphology was photographed 2 days after co-incubation (upper panel), and the GFP signals were detected under a fluorescent microscope (lower panel).

To investigate the interbacterial competition ability contributed by HSI-I and HSI-II, a GFP-expressing plasmid was introduced into each competitor bacterial strain indicated in Figure 1. For checking the role of each gene in the HSI-II cluster, *E. coli* MG1655 and *Psph* 1448a were chosen as representative competitors. Cells from overnight cultures of *Pst* DC3000 wild type, its derivatives, and competitor bacterial strains were collected, washed and adjusted to 1 × 10^7^ cfu/mL. Strains of *Pst* DC3000 were then mixed with each competitor in a 10:1 ratio, except for quantification of *Psph 1448a* surviving after co-incubation (a 1:1 ratio was used). Ten microliters of each mixture were spotted onto 0.22 μm nitrocellulose membranes placed on KBM plates and incubated for 2 days at 28°C. The GFP signals for each colony were recorded under a fluorescent stereomicroscope (Nikon Instruments Inc., Melville, NY, United States). The number of each bacterial strain in a mixed culture was counted by a method described previously (Hood et al., 2010).

### Live/dead Staining of Bacterial Cells

Interbacterial competition assay between *Pst* DC3000 and *E. coli* MG1655 was performed as described above. Two days after incubation at 28°C, survival rate of *Pst* DC3000 and *E. coli* MG1655 in a mixed (*Pst*: *E. coli* = 1:1) or pure culture was determined with a BacLight Bacterial Viability Kit (Invitrogen, Grand Island, NY, United States) as described previously (Hood et al., 2010); survival rate (%) was calculated as [(live cells/total cells) × 100].

### Analysis of Secreted Proteins

Overnight cultures of different *Pst* DC3000 strains were subcultured into fresh KBM at OD_600_ = 0.1. After growing to OD_600_ = 0.8 at 28°C, culture media and bacterial cells were separated by centrifugation at 4,000 rpm, and then the supernatants were passed through 0.22 μm syringe filters. Secreted proteins were precipitated from culture media by using 10% trichloroacetic acid and washed with ice-cold acetone twice. The cell pellets and secreted proteins underwent Western blot analysis with anti-Hcp2 and anti-RpoA (RNA polymerase alpha subunit) antisera as primary antibodies and horseradish peroxidase (HRP)-conjugated anti-rabbit IgG as the secondary antibody. Signals of the target proteins were detected by using Immobion Chemiluminescent HRP substrate (Millipore Corp., Bilerica, MA, United States) with a G: BOX system (Syngene, Frederick, MD, United States).

## Results

### Deletion of the HSI-II but Not HSI-I Gene Cluster Decreased Fluorescence Signals of Several Plant-Associated Gram-Negative Bacteria Harboring GFP Markers

Previous study showed that *Pst* DC3000 confers growth advantages over *E. coli* and yeast in a *hcp2-*dependent manner (Haapalainen et al., 2012). Which T6SS gene cluster (HSI-I and HSI-II) is responsible for this anti-bacterial and anti-yeast activities remains unclear. Moreover, whether T6SS is required for *Pst* DC3000 to survive in its natural niche full of other plant-associated bacteria and how HSI-I and HSI-II gene clusters are involved in T6SS apparatus assembly and effector delivery in *Pst* DC3000 remain unknown. To attempt to answer these questions, we first deleted HSI-I and HSI-II gene clusters singly and in combination to examine whether interbacterial competition activities of *Pst* DC3000 were altered. For a quick screen, a plasmid carrying constitutively expressed green fluorescence protein (*gfp*) was transformed into the selected plant-associated bacteria, including *Agrobacterium tumefaciens* C58, *Dickeya dadantii* CAS9, *Pst* T1, *P. s.* pv. *syringae* (*Pss*) 61, *P. savastanoi* pv. *phaseolicola* (*Psph*) 1448A, *P. s.* pv. *tabaci* (*Psta*) 11528, *P. viridiflava*, *P. putida* A7, and *Xanthomonas euvescatoria* XvT147 as well as *E. coli* MG1655. These competitor bacterial strains were then individually mixed with cells of the *Pst* DC3000 wild type and derived mutants at a 10:1 ratio. Two days after co-incubation on KBM at 28°C, changes in GFP intensity were observed under a fluorescent stereomicroscope. As shown in Figure 1, regardless of strain of *Pst* DC3000 co-incubated, the colonies of *A. tumefaciens* C58 or *P. viridiflava* looked similar under visible or UV light in terms of GFP fluorescence signal. However, the GFP signal intensity was decreased to various degrees when the bacterial competitors *D. dadantii* CAS9, *E. coli* MG1655, *Psph* 1448a, *Pss* 61, *Psta* 11528, *Pst* T1, and *X. euvesicatoria* XvT147 were co-incubated with *Pst* DC3000 wild type and ΔHSI-I versus incubation alone or co-incubation with ΔHSI-II and ΔHSI-I/II. Unexpectedly, GFP signal was not detected in *P. putida* A7; however, smaller colonies could be observed when *P. putida* A7 was co-incubated with the *Pst* DC3000 wild type and ΔHSI-I, which suggests their ability to inhibit growth or spreading of *P. putida* A7 (Figure 1). Therefore, deletion of HSI-II but not HSI-I could decrease the fluorescence signal of various plant-associated bacteria harboring GFP markers, which suggests that HSI-II is responsible for interbacterial competition activity of *Pst* DC3000 against *E. coli* and plant-associated bacteria. Further characterization focusing on HSI-II gene cluster should help understand how T6SS confers growth advantages over other Gram-negative bacteria in *Pst* DC3000.

### HSI-II Gene Cluster Is Expressed Under Both Nutrient-Rich and Poor Conditions

**FIGURE 2:**
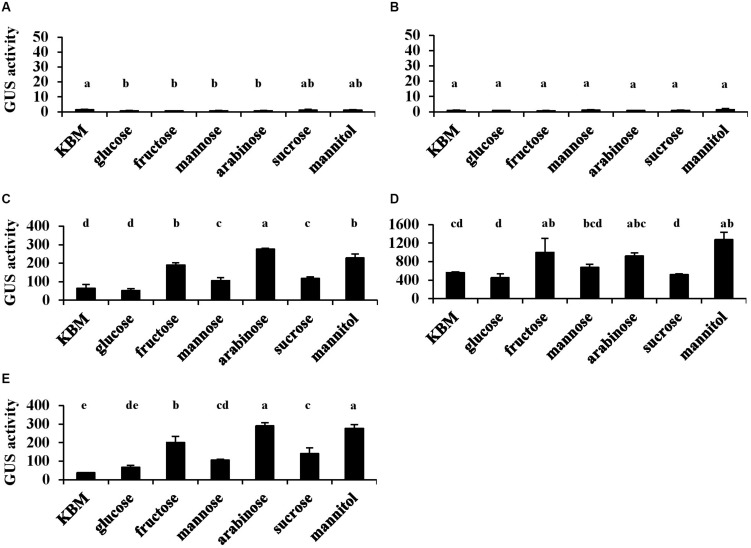
The activities of five putative promoters located in the HSI-I and HSI-II gene clusters of *Pst* DC3000. Each *Pst* DC3000 strain carrying *uidA* controlled by the 500 bp upstream sequence of *PSPTO_2542* (**A**; P*HSI-I*), *PSPTO_2539* (**B**; P*hcp1* in HSI-I), *PSPTO_5427* (**C**; P*HSI-II-i*), *PSPTO_5434* (**D**; P*HSI-II-ii*), or *PSPTO_5435* (**E**; P*hcp2* in HSI-II) was cultured in King’s B medium (KBM) or *hrp*-minimal salt supplemented with different carbohydrates as indicated. Fold change of the GUS activity was calculated as described in section “Materials and Methods.” Data are mean ± SD (*n* = 3). Data analysis involved one-way ANOVA and Tukey’s HSD test. Different letters indicate significant difference between groups (*p* < 0.05). This experiment was performed three times with similar results.

The type III secretion system of *Pst* DC3000 was induced only when cells were grown under a condition mimicking apoplastic growth *in planta* (i.e., in HMM at 18°C). To investigate how T6SS is regulated in *Pst* DC3000, each putative promoter in HSI-I and HSI-II clusters was transcriptionally fused to the *uidA* gene for a GUS reporter assay to monitor the promoter activity under different carbon sources. Consistent with a previous study (Sarris et al., 2010), the two promoters in HSI-I (Figures 2A,B) showed no activity when *Pst* DC3000 was grown in King’s medium B or HMM supplemented with all carbon sources tested, but three promoters in the HSI-II cluster (Figures 2C–E) were expressed under all tested conditions. The promoter region of the *tssB*/*C*-containing operon was expressed approximately fourfold higher than the other two operons (Figure 2D). Although the HSI-II gene cluster appears to be expressed constitutively in both minimal and nutrient-rich media, its expression was modulated by distinct carbon sources with fructose, arabinose and mannitol, in particular, conferring higher expression.

### HSI-II Gene Cluster Contains Three Transcriptional Units

**FIGURE 3:**
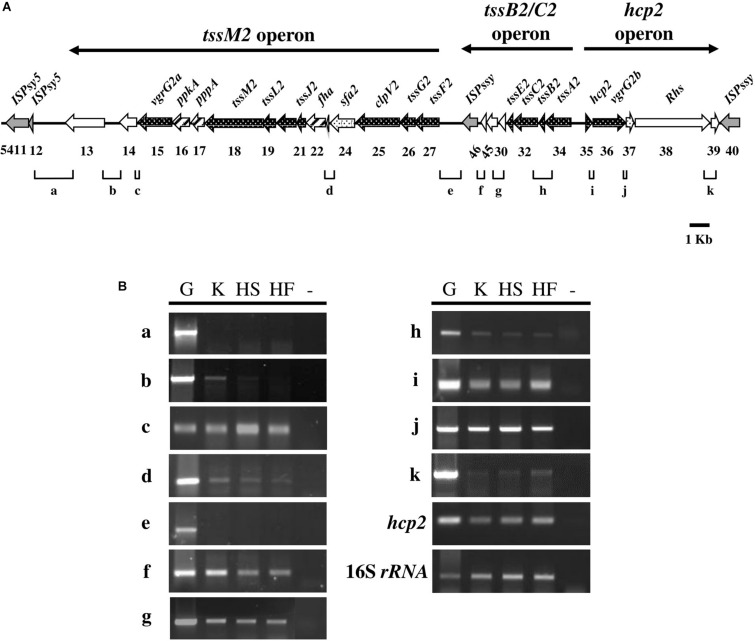
Transcript mapping of the HSI-II gene cluster in *Pst* DC3000. **(A)** Schematic diagram exhibiting 27 genes (*PSPTO_5413* to *PSPTO_5439*) of the HSI-II gene cluster and the intergenic regions “a∼k” analyzed in **(B)**. Black arrows indicate open reading frames (ORFs) orthologous to the core T6SS components (names are given above and gene ID numbers are shown below) and genes coding for putative transposases are shaded gray. Dotted arrows represent ORFs whose orthologs can be found in the HSI-I gene cluster. Arrows with diagonal lines represent ORFs orthologous to the regulatory T6SS components. The arrows above the diagram represent the extent of the three transcripts/operons, namely *tssM2*, *tssB2/C2*, and *hcp2* operons. **(B)** Semi-quantitative RT-PCR analysis of transcriptional units in the HSI-II gene cluster. Three culture media were used to investigate the expression of each intergenic region. K, KBM; HS, Hrp minimal salt (HMS) supplemented with sucrose; HF, HMS supplemented with fructose. The expression of *hcp2* was a positive control and 16S rRNA was an internal control. “G” and “-” indicate genomic DNA and a negative control, respectively. This experiment was repeated using three individual RNA samples with similar results.

In the *Pseudomonas* Genome Database, the HSI-II gene cluster was annotated as a region containing 24 genes, from PSPTO_5415 to PSPTO_5438, divided into six transcriptional units: PSTPO_5415-5427, PSPTO_5428, PSPTO_5645-5646, PSPTO_5430-5434, PSPTO_5435, and PSPTO_5436-5438. Information for each gene is provided in Table 1. In addition to using the *tss*/*tag* nomenclature system, gene names were also based on H2-T6SS of *Pseudomonas aeruginosa* PAO1, to which the HSI-II gene cluster is homologous. The putative transposase genes (PSPTO_5411, 5412, 5428, and 5440) were in or flanked this region, which infers possible horizontal gene transfer events (Figure 3A), and thus PSPTO_5413, PSPTO_5414, and PSPTO_5439 may also be part of this cluster. Moreover, the predicted functions of these genes based on use of HHPred and Phyre2 programs also signified their relevance to the T6SS secretion or interbacterial competition function: PSPTO_5413 encodes a putative protein with EF-hand, colicin and peptidase domains; PSPTO_5414 is a putative lipoprotein; and PSPTO_5439 was predicted as an Rhs domain-containing toxin (also summarized in Table 1). To verify the composition of the HSI-II gene cluster, the extent of the transcriptional units in this region was elucidated by using a series of primers to amplify the regions between two predicted transcriptional units (Figure 3A and Supplementary Table S3). When cells were grown in both rich and minimal media, we detected all transcripts except those containing intergenic regions between PSPTO_5412 and PSPTO_5413, and PSPTO_5427, and PSPTO_5646 could not be detected (Figure 3Ba,e). These data indicate that PSPTO_5413, PSPTO_5414, and PSPTO_5439 are indeed part of the HSI-II cluster: PSPTO_5413 and 5414 could be expressed with PSPTO_5415-5427 as an operon, PSPTO_5428 and PSPTO_5645-5646 belong to PSPTO_5430-5434 operon, and PSPTO_5435 and PSPTO_5439 are members of PSPTO_5436-5438 operon. The three major transcriptional units were hereafter named *tssM2* (PSPTO_5427 to PSPTO_5413), *tssB2/C2* (PSPTO_5434 to PSPTO_5428), and *hcp2* (PSPTO_5435 to PSPTO_5439) operons.

**TABLE 1:** Putative function of proteins encoded in the HSI-II gene cluster and their orthologs in the HSI-I gene cluster of *Pseudomonas syringae* pv. tomato DC3000.

Gene ID	Gene name^a^	Tss/Tag name^b^	Putative function^c^	Ortholog^d^
PSPTO_5413	–	–	EF-hand domain containing protein	–
PSPTO_5414	–	–	TPR repeat-containing lipoprotein	–
**PSPTO_5415**	*vgrG2a*	*tssI2a*	Rhs element Vgr protein	PSPTO_2538 (57.2)
PSPTO_5416	*ppkA*	*tagE*	Serine/threonine protein kinase	–
PSPTO_5417	*pppA*	*tagG*	Serine/threonine phosphoprotein phosphatase	–
**PSPTO_5418**	*icmF2*	*tssM2*	ImcF-related	PSPTO_2554 (57.8)
**PSPTO_5419**	*dotU2*	*tssL2*	DotU family; membrane protein	PSPTO_2553 (51.9)
**PSPTO_5420**	*hsiJ2*	*tssK2*	Hypothetical protein	PSPTO_2552 (62.1)
**PSPTO_5421**	*Lip2*	*tssJ2*	Lipoprotein	PSPTO_2551 (52.1)
PSPTO_5422	*fha*	*tagH*	FHA domain-containing protein	–
PSPTO_5423	*orfX*	–	Unknown function	–
PSPTO_5424	*sfa2*	*vasH2*	σ^54^-dependent transcription factor	PSPTO_2549 (55.4)
**PSPTO_5425**	*clpV2*	*tssH2*	clpB protein; AAA + ATPase	PSPTO_2548 (67.9)
**PSPTO_5426**	*hsiH2*	*tssG2*	Unknown function	PSPTO_2547 (63.9)
**PSPTO_5427**	*hsiG2*	*tssF2*	Hypothetical protein	PSPTO_2546 (55.3)
PSPTO_5646	–	–	Ferrodoxin-like protein	–
PSPTO_5645	–	–	Endonuclease-like protein	–
PSPTO_5430	–	–	PAAR motif-containing protein	–
**PSPTO_5431**	*hsiF2*	*tssE2*	GPW/gp25 family protein	PSPTO_2545 (49.6)
**PSPTO_5432**	*hsiC2*	*tssC2*	Subunit for the outer tube	PSPTO_2544 (83.9)
**PSPTO_5433**	*hsiB2*	*tssB2*	Subunit for the outer tube	PSPTO_2543 (71.4)
**PSPTO_5434**	*hsiA2*	*tssA2*	ImpA family	PSPTO_2540 and PSPTO_2542 (48.6)^e^
**PSPTO_5435**	*hcp2*	*tssD2*	Secreted protein Hcp	PSPTO_2539 (51.2)
**PSPTO_5436**	*vgrG2b*	*tssI2b*	Rhs element Vgr protein	PSPTO_2538 (60.4)
PSPTO_5437	–	–	DUF4123 superfamily	
PSPTO_5438	–	–	Rhs family protein	
PSPTO_5439	–	–	Rhs-repeat containing toxin	–

*^*a*^Gene names were proposed on the basis of the nomenclature system for the Pseudomonas aeruginosa H2-T6SS gene clusters and the system used in Sarris et al. (2010). ^*b*^Gene names are based on the universal tss nomenclature system proposed by Shalom et al. (2007). ^*c*^The putative function of each gene product is based on the functional predictions from Interpro, Pseudomonas Ortholog Group members described in the Pseudomonas Genome Database (http://www.pseudomonas.com; Winsor et al., 2016) and Pfam and Phyr2 prediction. Genes coding for core components of T6SS are in bold. ^*d*^Ortholog genes found in the HSI-I gene cluster of P. s. pv. tomato DC3000. Numbers in parenthesis are amino acid sequence identity (%) between two orthologous gene products. ^*e*^The DNA sequence encoding an hsiA2 ortholog in HSI-I is interrupted by PSPTO_2541, an ISPssy transposase. The amino acid sequence identity was calculated by using that for PSPTO_2540 and PSPTO_2542.*

### Deletion of Most Genes in HSI-II Gene Cluster Changed Hcp2 Secretion

**FIGURE 4:**
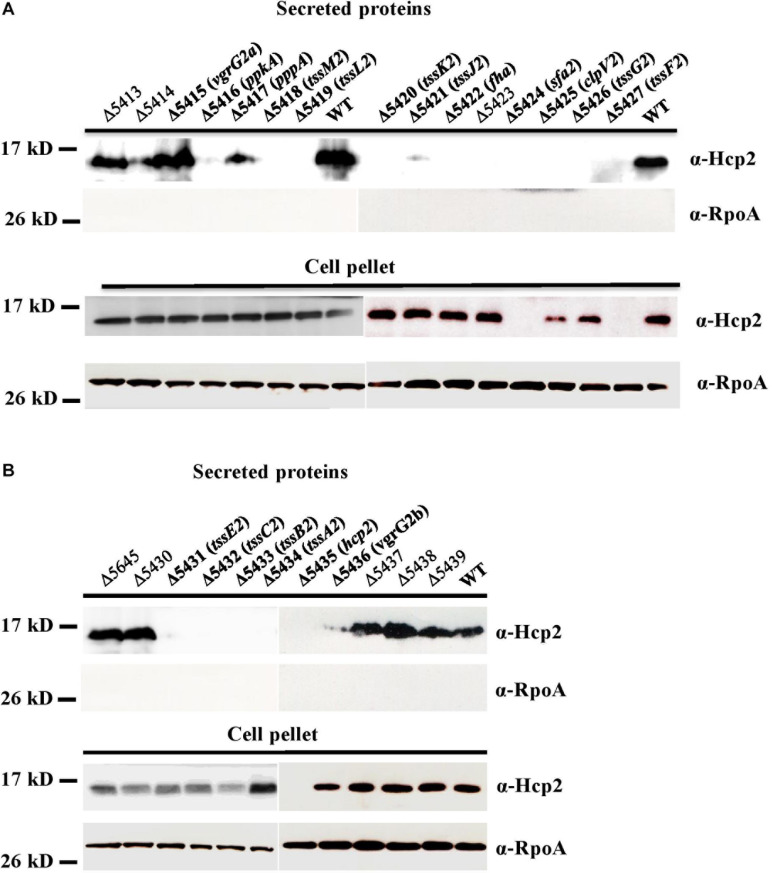
Secretion assay differentiates the role of each gene in the HSI-II gene cluster. *Pst* DC3000 wild type and mutants with deletion of each gene in the *tssM2* operon **(A)** and in the *tssB2/C2* and *hcp2* operons **(B)** were grown in KBM at 28°C from OD_600_ 0.1 to 0.8. Western blot analysis was used to detect Hcp2 in the culture media (secreted proteins) and cell pellets with anti-Hcp2 antiserum. RpoA was an internal control for cytoplasmic proteins (equal loading) and a negative control for secreted proteins.

In addition to the genes coding for the core components of T6SS, the putative attributes of other non-conserved genes in the HSI-II gene cluster for T6SS functions are listed in Table 1. To determine whether those genes are critical for T6SS functions, we constructed in-frame deletion mutants for each gene in the HSI-II cluster and obtained single mutants for 26 genes, except PSPTO_5646. Because Hcp2 is the only marker protein of T6SS secretion function detected so far in *Pst* DC3000 (Haapalainen et al., 2012), Hcp2 secretion was analyzed to assess the effect of these mutants on T6SS secretion. Cellular Hcp2 proteins accumulated in most of the mutants, except no or few Hcp signals were detected in cell pellets of Δ5435 (Δ*hcp2)*, Δ5424 with deletion of *sfa2* encoding a σ^54^-dependent transcriptional regulator, and Δ5427 with deletion of *tssF2* (Figures 4A,B, “Cell Pellet”). Regardless of normal Hcp2 levels inside the cells, deletion of genes coding for core components affected the ability of *Pst* DC3000 to secrete Hcp2. Although both PSPTO_5415 and PSPTO_5436 encode VgrG homologs, only deletion of PSPTO_5436 resulted in loss of Pst DC3000 ability to secrete Hcp2. Deletion of PSPTO_5416 and 5422, encoding the regulatory components PpkA and Fha, respectively, also impaired Hcp2 secretion. However, repression of Hcp2 secretion by PSPTO_5417, coding for a PppA ortholog, was not observed in *Pst* DC3000 (Mougous et al., 2007; Fritsch et al., 2013; Lin et al., 2014). In addition to the core structural genes (*tssA2-M2*) and positive regulatory genes (*ppkA* and *fha*), Δ5423, representing deletion of an accessory gene *orfX* with unknown function, also impaired Hcp2 secretion (Figures 4A,B, “Secreted protein”).

### Deletion of Most Genes in the HSI-II Gene Cluster Reduced the Interbacterial Competition Ability of *Pst* DC3000

**FIGURE 5:**
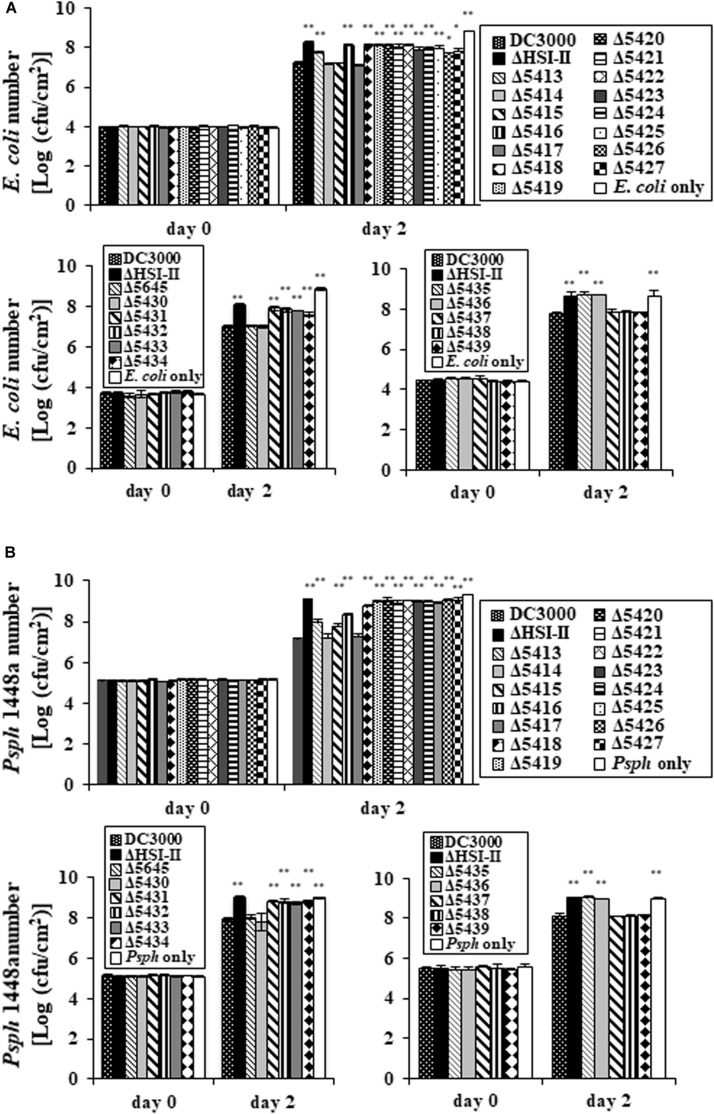
Deletion of each gene in the HSI-II gene cluster affects the interbacterial competition activity of *Pst* DC3000. After co-incubation with GFP-expressing *E. coli* MG1655 or *Psph* 1448a for 2 days, the competition ability of each mutant was evaluated by calculating the bacterial number of *E. coli* **(A)** and *Psph* 1448a **(B)**. “*E. coli* only” and “*Psph* only” indicate bacterial numbers without co-incubation with any *Pst* DC3000 strain. Data analysis involved Student’s *t*-test. Data are mean ± SD (*n* = 3) *P < 0.05; **P < 0.01 compared with the wild type. This experiment was conducted three times with similar results.

Deletion of most genes in the *tssM2* operon (PSPTO_5414 to PSPTO_5427) reduced the ability of *Pst* DC3000 to compete against several plant-associated Gram-negative bacteria (Figure 1). This phenomenon was also observed when the *hcp2* operon was deleted, so the contribution of the *tssM2* and *hcp2* operons to interbacterial competition was similar on the basis of bacterial counts after co-incubation (Supplementary Figure S2). Among the Gram-bacteria tested in Figure 1, *Psph* 1448a and *E. coli* MG1655 were selected as representatives for competitors with and without functional T6SS, respectively, and used to determine the interbacterial competition ability of each single mutant of the HSI-II gene cluster. *Pst* DC3000 growth did not differ when mutants were co-incubated with *Psph* 1448a or *E. coli* MG1655 or incubated alone (Supplementary Figure S3). However, all mutants found impaired in Hcp secretion lost or reduced their ability to cause growth inhibition of the competitors, whereas the mutants with slightly reduced or wild-type levels of Hcp2 secretion (Δ5414, Δ5417, Δ5645, Δ5430, Δ5437, Δ5438, and Δ5439) retained the same interbacterial competition activity (Figure 5). Of note, deletion of PSPTO_5415 reduced its ability to antagonize *Psph* 1448a but retained the wild-type antibacterial activity to *E. coli* MG1655 (Figures 5A,B). Hence, mutants with malfunction in Hcp2 secretion did not always alter their ability to inhibit growth of *E. coli* MG1655 or *Psph* 1448a. Furthermore, PSPTO_5413, which is not required for Hcp2 secretion, was critical for the competition ability of *Pst* DC3000, which suggests that PSPTO_5413 may be a T6SS effector targeting competitors for growth fitness.

### HSI-II–Reduced Competitor Growth Is a Cell Contact-Dependent and Bacteriostatic Effect

**FIGURE 6:**
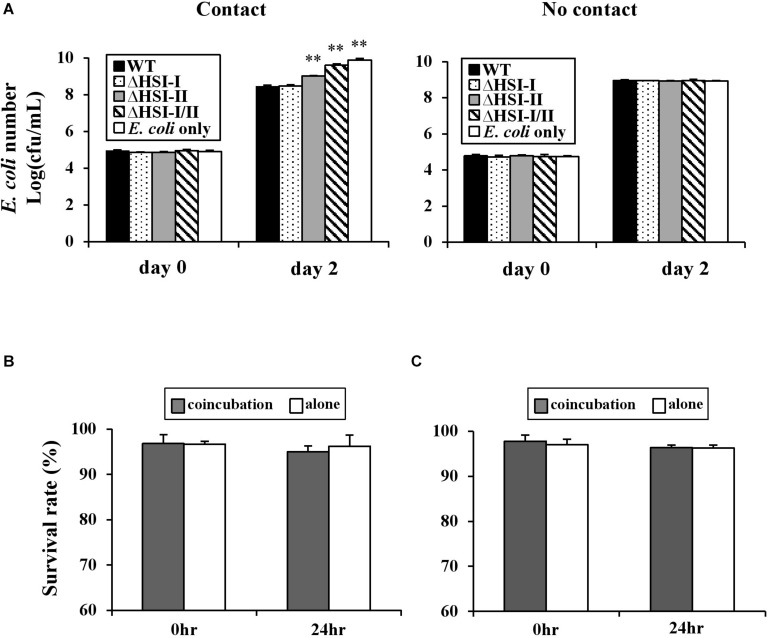
HSI-II-mediated antibacterial toxicity depends on cell contact and is a bacteriostatic effect. **(A)** The GFP-labeled *Pst* DC3000 and its derivative mutants were co-incubated with (contact) or separated from the test strain *E. coli* MG1655 by a 0.22 μm filter membrane (no contact). Two days after incubation at 28°C, *E. coli* numbers in each treatment were measured. “*E. coli* only” indicates bacterial number without co-incubation with any *Pst* DC3000 strain. Survival rate of *Pst* DC3000 **(B)** and *E. coli* K-12 MG1655 **(C)** in a mixed (*Pst*: *E. coli* = 10:1) or pure culture calculated with a BacLight Bacterial Viability Kit. Data are mean ± SD (n = 3) and were analyzed with Student’s *t*-test. ^∗∗^*P* < 0.01 compared with the wild type. This experiment was conducted three times with similar results.

T6SS-mediated interbacterial competition is likely mediated in a cell contact-dependent manner as experimentally demonstrated in *Aeromonas hydrophila*, *Burkholderia thailandensis*, *P. aeruginosa*, and *V. cholerae* (Russell et al., 2014). To determine whether the observed interbacterial competition activity of *Pst* DC3000 requires cell–cell contact, with a competition assay, we placed a 0.22 μm nitrocellulose membrane between *E. coli* MG1655 and *Pst* DC3000. When *E. coli* and *Pst* DC3000 were mixed together without a membrane, HSI-II-dependent inhibition of *E. coli* MG1655 growth was detected (Figure 6A, “Contact”). In contrast, this HSI-II-dependent reduction of *E. coli* cell count was not detectable when a membrane was placed between *E. coli* MG1655 and *Pst* DC3000 (Figure 6A, “No contact”). The same results were obtained when different competing bacteria were tested (data not shown). Also, *E. coli* growth did not decrease much, which suggests that the HSI-II-mediated reduction of competitor growth may be a weak bacteriocidal or a bacteriostatic effect. To answer this question, we calculated the survival rates of both *Pst* DC3000 and *E. coli* MG1655 during co-inoculation by using a BacLight Bacterial Viability Kit. Survival rates of *Pst* DC3000 (Figure 6B) and *E. coli* MG1655 (Figure 6C) did not differ when the two bacteria were co-incubated with each other or incubated alone. Thus, cell-to-cell contact was required for HSI-II–mediated toxicity of *Pst* DC3000, and this interbacterial competition activity was a bacteriostatic effect.

### PSPTO_5424 Encodes a Transcriptional Regulator Controlling the Expression of the HSI-II Gene Cluster

**FIGURE 7:**
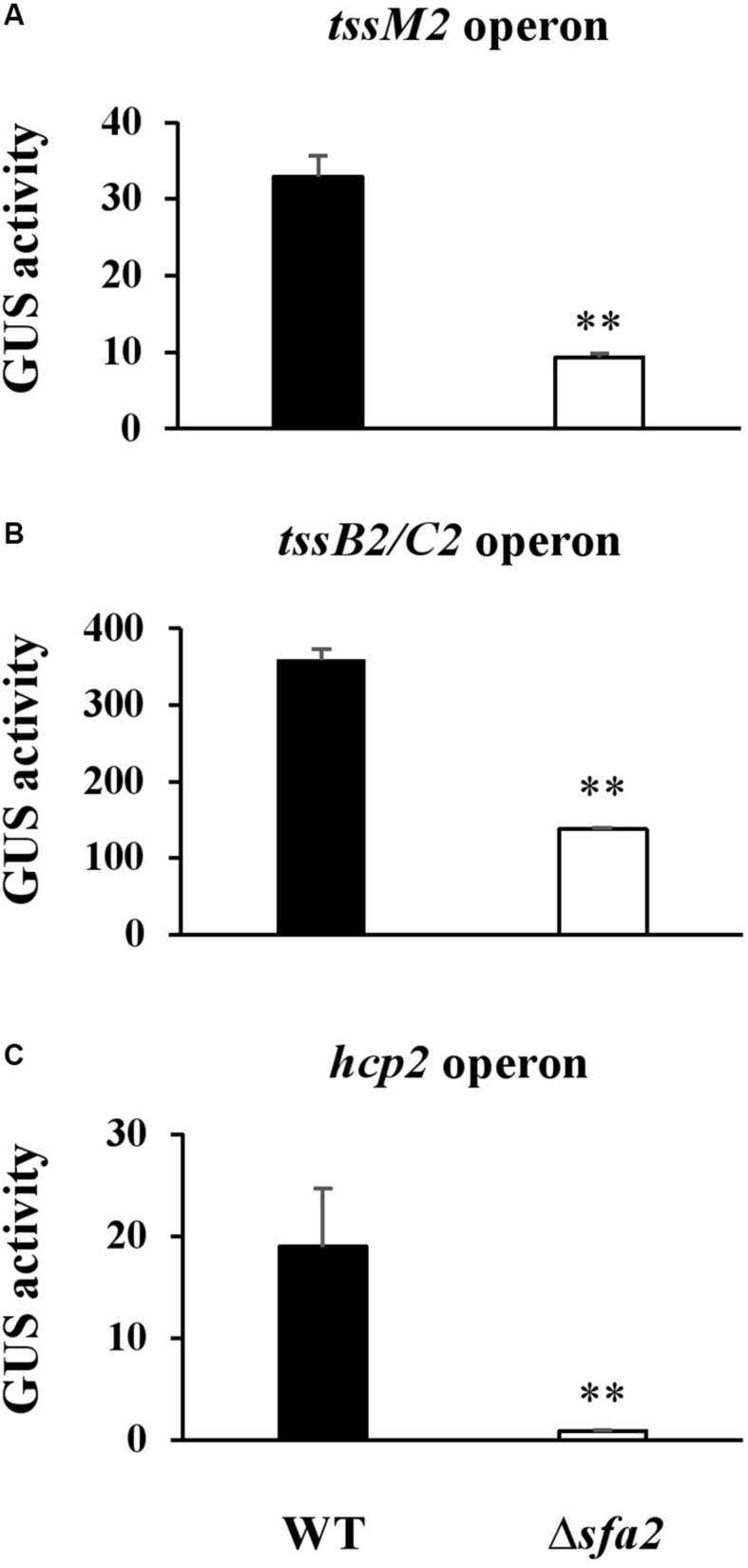
Sfa2 positively regulates the expression of three operons in the HSI-II gene cluster. *Pst* DC3000 or Δ5424 (Sfa2) carrying *uidA* controlled by the 500 bp upstream sequence of **(A)** *tssM2*, **(B)** *tssB2/C2* or **(C)** *hcp2* operon were cultured in KBM from OD_600_ = 0.1 to 0.8 before bacterial cells were collected for determining GUS activity. Fold change of the GUS activity was calculated as described in section “Materials and Methods.” Data are mean ± SD (*n* = 3) ^∗∗^*P* < 0.01 compared with the wild type. This experiment was conducted three times with similar results.

PSPTO_5424 was annotated as a *Sfa* ortholog, a putative σ^54^-dependent transcriptional regulator, and could control T6SS gene expression in several strains of *V. cholorae* and *P. aeruginosa* PAO1 (Pukatzki et al., 2006; Sana et al., 2013). Data in Figure 4 implied that Sfa2 mediates the expression of Hcp2; however, whether the other two operons in the HSI-II gene cluster are also regulated by Sfa2 remain uncharacterized. Thus, we used GUS reporter assay to check the promoter activities of *tssM2* and *tssB2/C2* operons in the presence or absence of Sfa2. Similar to the *hcp2* operon, the promoter activities of these two operons were decreased in Δ*sfa2* as compared with the wild type, which indicates that Sfa2 not only affects the expression of *hcp2* but also participates in regulating the other two operons in the HSI-II gene cluster (Figure 7).

## Discussion

As an archetypical phytopathogenic bacterium, *Pst* DC3000 has been studied extensively in terms of its biology, ecology and evolution. Genomic analyses of T6SS loci in all *Pseudomonas* spp. sequenced to date and from many divergent bacterial strains revealed several unique characteristics of T6SS in *Pst* DC3000: (1) it is one of the two strains (i.e., *Pst* DC3000 and *P. putida* KT2440) with two copies of a similar T6SS cluster (cluster 1.1 in Barret et al., 2011); (2) the HSI-II gene cluster contains a complete set of T6SS core and regulatory components, whereas HSI-I lacks genes encoding Fha, PphA, and PpkA; (3) the gene coding for a core component, HsiA/TssA, is interrupted by a transposase in the HSI-I gene cluster, probably leading to loss-of-function of this locus; (4) unlike the other *P. syringae* strains, *Pst* DC3000 does not possess the cluster 4B T6SS locus (Sarris et al., 2010; Barret et al., 2011). In this study, we demonstrated that the HSI-II cluster of *Pst* DC3000 encodes a functional T6SS conferring secretion and interbacterial competition activities. In contrast, no HSI-I activity could be detected in all assays performed, which again shows that HSI-I is inoperative. Although the HSI-II gene cluster is phylogenetically related to H2-T6SS of *P. aeruginosa* PAO1 (Bingle et al., 2008; Boyer et al., 2009), it does not contribute to virulence (Haapalainen et al., 2012), only interbacterial competition.

Unlike the type III secretion system of *Pst* DC3000, which is activated only *in planta* or under conditions mimicking apoplastic fluid (Huynh et al., 1989; Rico and Preston, 2008), all three operons in the HSI-II gene cluster were expressed constitutively in both rich and minimal media, although different carbon sources could affect their expression level. T6SS-mediated antibacterial toxicity may allow pathogens to secure their niches by competing with other bacteria in the environment or during infection, and different expression patterns of T6SS may reflect strategies of how to use the T6SS apparatus. For instance, *V. cholerae* uses T6SS as an offensive weapon, so constitutive expression of T6SS is usually prevalent in the environmental isolates, which are frequently exposed to the competitors or predators (Unterweger et al., 2012). However, T6SS serves as a defensive tool in *P. aeruginosa*, which detects the threat from other T6SS^+^ bacteria, and thus T6SS is stimulated when T6SS dueling occurs (Basler et al., 2013). Nevertheless, attempts to evaluate the growth fitness of the *Pst* DC3000 wild type and ΔHSI-II mutant using field soils containing abundant microorganisms was not successful because very low rates of both *Pst* DC3000 and its T6SS mutant were recovered from field soils after incubation for only 1 day (data not shown). In fact, records have linked higher survival of *Pst* to tomato seeds and host debris but not field soils (McCarter et al., 1983). With the weak performance of the HSI-II-mediated bacteriostatic effect (demonstrated by less reduction of competitor numbers in Figure 5), the continuous presence of other microorganisms during its life cycle likely puts much selective pressure on *Pst* DC3000, leading to its evolution into an entity constitutively expressing T6SS to maintain its population in the environment before host plants are present. The antibacterial activity is so weak that HSI-II–mediated growth advantage could only be detected when the circumstance is not as complicated as field soils or the bacterial concentration is higher, namely in a competition assay, seeds, or host debris.

T6SS effectors responsible for antibacterial activity have been identified in many bacteria, and many are encoded within the T6SS loci, which implies that their secretion strongly depends on the associated T6SS apparatus (Miyata et al., 2011; Russell et al., 2012; Ma et al., 2014; Whitney et al., 2014; Yang et al., 2015). Bioinformatics analysis predicted PSPTO_5413, PSPTO_5438, and PSPTO_5439 as VgrG-associated effectors (Hachani et al., 2014; Whitney et al., 2014; Liang et al., 2015), whereas PSPTO_5645/5646 could be effector/immunity pairs linked to the PAAR protein (PSPTO_5430) (Table 1). The antibacterial toxins are usually produced concurrently with their cognate immunity proteins to avoid self-intoxication (Russell et al., 2012). Thus, genes coding for immunity proteins are essential for protection against T6SS-dependent attack of neighboring sister cells, and mutations in immunity genes could be lethal in T6SS^+^ strains (Dong et al., 2013). That we could not obtain mutants of PSPTO_5646 also suggests that it could be the cognate immunity protein of PSPTO_5645. However, deletion of PSPTO_5645 has no effect on interbacterial competition ability, which suggests that PSPTO_5645 may not function as a potent antibacterial toxin or the chosen competitors (*E. coli* and *Psph*) are not the targets of PSPTO_5645.

Among four putative effector genes identified in this study, only deletion of PSPTO_5413 caused reduced interbacterial competition activity against *E. coli* and *Psph.* The evidence that PSPTO_5413 plays no role in mediating Hcp2 secretion is consistent with its accessory role not related to T6SS assembly. However, constitutively expressing PSPTO_5413 in *E. coli* MG1655 or *Psph*1448a did not reveal growth inhibition (data not shown). The lack of toxicity in the target cells may suggest that additional factors(s) from *Pst* DC3000 may be required for toxicity of PSPTO_5413, or subcellular location of ectopically expressed PSPTO_5413 may not be the site of its mode of action. Analysis of the amino acid sequence of PSPTO_5413 revealed the presence of an EF-hand motif and a domain highly similar to the catalytic domain of the antimicrobial peptidase lysostaphin. EF-hand motifs can be found in calcium-binding proteins, acting in Ca^2+^ homeostasis or calcium signaling (Yanez et al., 2012). Lysostaphin is a glycylglycine endopeptidase identified in *Staphylococcus simulans*, which can cleave the peptide bond between the third and fourth glycine residues of the pentagycine cross-link in the cell wall of *S. aureus* (Sabala et al., 2014). Despite limited information on the roles of EF-hand calcium-binding in prokaryotes (Michiels et al., 2002; Dominguez et al., 2015), the structural and biochemical characteristics of the lysin LysGH15 from Staphylococcal phage GH15 shed light on the possible mechanism underlying the toxic effect of PSPTO_5413 (Gu et al., 2014). PSPTO_5413 is likely a cell wall-targeting effector, whose activity is modulated dependent on calcium concentration via the function of the EF-hand motif. Because of all these characteristics and the presence of PSPTO_5413 orthologs in only a few *Pseudomonas* spp., this gene is unique and worthy of further dissection to clarify how PSPTO_5413 is involved in the interbacterial competition activity of *Pst* DC3000 and whether PSPTO_5414, coding for a lipoprotein, might be its cognate immunity protein.

In searching for putative functions of each gene in the HSI-II gene cluster, we identified proteins containing DUF4123 (PSPTO_5437) and recombination hotspot (Rhs) domains (PSPTO_5438 and 5439) (Table 1). DUF4123, together with DUF1759 and DUF2169, has been characterized as a domain associated with the chaperone/adaptor proteins of the T6SS cargo effectors and is required for effector delivery as well as the bacterial killing activity (Unterweger et al., 2015; Bondage et al., 2016; Cherrak et al., 2019). This conserved domain has been used to identify some novel T6SS effectors (Liang et al., 2015; Zepeda-Rivera et al., 2018). However, *E. coli* MG1655 or *Psph* 1448a co-incubated with Δ5437, Δ5438, and Δ5439 did not reduce bacterial growth, which suggests that these three genes do not participate in a growth advantage of *Pst* DC3000 over *E. coli* MG1655 or *Psph* 1448a (Figure 5). Besides PSPTO_5437, three DUF4123-containing proteins in *Pst* DC3000 are PSPTO_2537, PSPTO_3483, and PSPTO_3850. PSPTO_2537 is located in the HSI-I gene cluster, whose expression cannot be detected thus far. PSPTO_3483 and PSPTO_3850 are both located in an orphan VgrG island. Of note, genes coding for putative toxins are also present downstream of PSPTO_3483 and PSPTO_3850. Thus, future work will focus on understanding whether these proteins are involved in growth fitness of *Pst* DC3000 and can be uploaded onto cognate VgrG with the help of a DUF4123-containing chaperone encoded in the same island for successful delivery by T6SS.

Several reports have shown that the expression of genes coding for T6SS apparatus can be modulated by the σ^54^-dependent transcriptional regulators encoded within T6SS loci. In *P. aeruginosa*, Sfa2 negatively regulates H2-T6SS expression, but H3-T6SS expression is Sfa3-independent (Sana et al., 2013). In *V. cholerae* and *A. hydrophila*, the σ^54^-dependent transcriptional regulator VasH positively regulates the expression of *hcp* genes (Pukatzki et al., 2006; Suarez et al., 2008). Although the HSI-II gene cluster is phylogenetically related to H2-T6SS of *P. aeruginosa*, the regulatory activity of Sfa2 (PSPTO_5424) in *Pst* DC3000 is opposite to that of Sfa2 in *P. aeruginosa*. Discovery of effector proteins is important for understanding the mechanism underlying T6SS functions. Use of a *vasH* mutant expressing VasH under the control of an arabinose-inducible promoter revealed an effector VasX encoded outside the T6SS locus (Miyata et al., 2011). Because *Pst* DC3000 and *V. cholerae* both positively control the expression of T6SS by a σ^54^-dependent transcriptional regulator, the same strategy may be applicable for discovery of additional T6SS effectors in *Pst* DC3000.

Using a systematic mutagenesis approach, we identified genes located in the HSI-II cluster that are required for T6SS activities, including accumulation or secretion of Hcp2, and interbacterial competition (summarized in Supplementary Table S4). Although more work is required to elucidate the effector functions and the mechanisms underlying HSI-II–mediated interbacterial competition, this study identified the regulatory elements and genes responsible for expression, assembly, and function of T6SS-mediated interbacterial of *Pst* DC3000. From this and previous studies, we now know that instead of providing virulence activity, HSI-II-T6SS is likely used as a defensive weapon to protect *Pst* DC3000 against bacteria sharing the same ecological niches to ensure sufficient inoculum for successful infection when encountering appropriate host plants. At present, secretome and transcriptome analyses are undergoing, which should help us identify the effectors and regulatory components of HSI-II-T6SS for better understanding the mechanistic and biological functions of HSI-II in *Pst* DC3000.

## Data Availability Statement

The raw data supporting the conclusions of this article will be made available by the authors, without undue reservation, to any qualified researcher.

## Author Contributions

C-FC and N-CL conceived and designed the experiments. C-FC, C-YL, Y-YL, Y-HS, K-YC, and N-CL performed the experiments and analyzed the data. C-FC and N-CL wrote the manuscript.

## Conflict of Interest

The authors declare that the research was conducted in the absence of any commercial or financial relationships that could be construed as a potential conflict of interest.

## Footnotes

1 http://www.pseudomonas.com

## References

[B1] Allsopp L. P. Bernal P. Nolan L. M. Filloux A. (2019). Causalities of war: the connection between type VI secretion system and microbiota. *Cell. Microbiol.* 22:e13153. 10.1111/cmi.13153 31872954 PMC7540082

[B2] Barret M. Egan F. Fargier E. Morrissey J. P. O’gara F. (2011). Genomic analysis of the type VI secretion systems in *Pseudomonas* spp.: novel clusters and putative effectors uncovered. *Microbiology* 157 1726–1739. 10.1099/mic.0.048645-0 21474537

[B3] Basler M. Ho B. T. Mekalanos J. J. (2013). Tit-for-tat: type VI secretion system counterattack during bacterial cell-cell interactions. *Cell* 152 884–894. 10.1016/j.cell.2013.01.042 23415234 PMC3616380

[B4] Basler M. Pilhofer M. Henderson G. P. Jensen G. J. Mekalanos J. J. (2012). Type VI secretion requires a dynamic contractile phage tail-like structure. *Nature* 483 182–186. 10.1038/nature10846 22367545 PMC3527127

[B5] Bernal P. Allsopp L. P. Filloux A. Llamas M. A. (2017). The *Pseudomonas putida* T6SS is a plant warden against phytopathogens. *ISME J.* 11 972–987. 10.1038/ismej.2016.169 28045455 PMC5363822

[B6] Bingle L. E. Bailey C. M. Pallen M. J. (2008). Type VI secretion: a beginner’s guide. *Curr. Opin. Microbiol.* 11 3–8. 10.1016/j.mib.2008.01.006 18289922

[B7] Bladergroen M. R. Badelt K. Spaink H. P. (2003). Infection-blocking genes of a symbiotic *Rhizobium leguminosarum* strain that are involved in temperature-dependent protein secretion. *Mol. Plant Microbe Interact.* 16 53–64. 10.1094/MPMI.2003.16.1.53 12580282

[B8] Blokesch M. (2015). Competence-induced type VI secretion might foster intestinal colonization by *Vibrio cholerae*: intestinal interbacterial killing by competence-induced *V. cholerae*. *Bioessays* 37 1163–1168. 10.1002/bies.201500101 26445388

[B9] Bondage D. D. Lin J. S. Ma L. S. Kuo C. H. Lai E. M. (2016). VgrG C terminus confers the type VI effector transport specificity and is required for binding with PAAR and adaptor-effector complex. *Proc. Natl. Acad. Sci. U.S.A.* 113 E3931–E3940. 10.1073/pnas.1600428113 27313214 PMC4941472

[B10] Borgeaud S. Metzger L. C. Scrignari T. Blokesch M. (2015). The type VI secretion system of *Vibrio cholerae* fosters horizontal gene transfer. *Science* 347 63–67. 10.1126/science.1260064 25554784

[B11] Boyer F. Fichant G. Berthod J. Vandenbrouck Y. Attree I. (2009). Dissecting the bacterial type VI secretion system by a genome wide *in silico* analysis: what can be learned from available microbial genomic resources? *BMC Genomics* 10:104. 10.1186/1471-2164-10-104 19284603 PMC2660368

[B12] Brunet Y. R. Zoued A. Boyer F. Douzi B. Cascales E. (2015). The type VI secretion TssEFGK-VgrG phage-like baseplate is recruited to the TssJLM membrane complex via multiple contacts and serves as assembly platform for tail tube/sheath polymerization. *PLoS Genet* 11:e1005545. 10.1371/journal.pgen.1005545 26460929 PMC4604203

[B13] Cherrak Y. Flaugnatti N. Durand E. Journet L. Cascales E. (2019). Structure and activity of the type VI secretion system. *Microbiol. Spectr.* 7. 10.1128/microbiolspec.PSIB-0031-2019 31298206 PMC10957189

[B14] Chow J. Mazmanian S. K. (2010). A pathobiont of the microbiota balances host colonization and intestinal inflammation. *Cell Host Microbe* 7 265–276. 10.1016/j.chom.2010.03.004 20413095 PMC2859213

[B15] Costa T. R. Felisberto-Rodrigues C. Meir A. Prevost M. S. Redzej A. Trokter M. (2015). Secretion systems in Gram-negative bacteria: structural and mechanistic insights. *Nat. Rev. Microbiol.* 13 343–359. 10.1038/nrmicro3456 25978706

[B16] Coulthurst S. (2019). The type VI secretion system: a versatile bacterial weapon. *Microbiology* 165 503–515. 10.1099/mic.0.000789 30893029

[B17] Dominguez D. C. Guragain M. Patrauchan M. (2015). Calcium binding proteins and calcium signaling in prokaryotes. *Cell Calcium* 57 151–165. 10.1016/j.ceca.2014.12.006 25555683

[B18] Dong T. G. Ho B. T. Yoder-Himes D. R. Mekalanos J. J. (2013). Identification of T6SS-dependent effector and immunity proteins by Tn-seq in *Vibrio cholerae*. *Proc. Natl. Acad. Sci. U.S.A.* 110 2623–2628. 10.1073/pnas.1222783110 23362380 PMC3574944

[B19] Durand E. Cambillau C. Cascales E. Journet L. (2014). VgrG, Tae, Tle, and beyond: the versatile arsenal of Type VI secretion effectors. *Trends Microbiol.* 22 498–507. 10.1016/j.tim.2014.06.004 25042941

[B20] Fritsch M. J. Trunk K. Diniz J. A. Guo M. Trost M. Coulthurst S. J. (2013). Proteomic identification of novel secreted antibacterial toxins of the *Serratia marcescens* type VI secretion system. *Mol. Cell. Proteomics* 12 2735–2749. 10.1074/mcp.M113.030502 23842002 PMC3790287

[B21] Gallagher S. R. (1992). *GUS Protocols: Using the GUS Gene as a Reporter of Gene Expression.* San Diego, CA: Academic Press.

[B22] Gu J. Feng Y. Feng X. Sun C. Lei L. Ding W. (2014). Structural and biochemical characterization reveals LysGH15 as an unprecedented “EF-hand-like” calcium-binding phage lysin. *PLoS Pathog.* 10:e1004109. 10.1371/journal.ppat.1004109 24831957 PMC4022735

[B23] Haapalainen M. Mosorin H. Dorati F. Wu R. F. Roine E. Taira S. (2012). Hcp2, a secreted protein of the phytopathogen *Pseudomonas syringae* pv. tomato DC3000, is required for fitness for competition against bacteria and yeasts. *J. Bacteriol.* 194 4810–4822. 10.1128/JB.00611-12 22753062 PMC3430304

[B24] Hachani A. Allsopp L. P. Oduko Y. Filloux A. (2014). The VgrG proteins are “a la carte” delivery systems for bacterial type VI effectors. *J. Biol. Chem.* 289 17872–17884. 10.1074/jbc.M114.563429 24794869 PMC4067218

[B25] Hachani A. Wood T. E. Filloux A. (2016). Type VI secretion and anti-host effectors. *Curr. Opin. Microbiol.* 29 81–93. 10.1016/j.mib.2015.11.006 26722980

[B26] Han Y. Wang T. Chen G. Pu Q. Liu Q. Zhang Y. (2019). A *Pseudomonas aeruginosa* type VI secretion system regulated by CueR facilitates copper acquisition. *PLoS Pathog.* 15:e1008198. 10.1371/journal.ppat.1008198 31790504 PMC6907878

[B27] Hood R. D. Singh P. Hsu F. Guvener T. Carl M. A. Trinidad R. R. (2010). A type VI secretion system of *Pseudomonas aeruginosa* targets a toxin to bacteria. *Cell Host Microbe* 7 25–37. 10.1016/j.chom.2009.12.007 20114026 PMC2831478

[B28] Huynh T. V. Dahlbeck D. Staskawicz B. J. (1989). Bacterial blight of soybean: regulation of a pathogen gene determining host cultivar specificity. *Science* 245 1374–1377. 10.1126/science.2781284 2781284

[B29] Liang X. Moore R. Wilton M. Wong M. J. Lam L. Dong T. G. (2015). Identification of divergent type VI secretion effectors using a conserved chaperone domain. *Proc. Natl. Acad. Sci. U.S.A.* 112 9106–9111. 10.1073/pnas.1505317112 26150500 PMC4517263

[B30] Lin J. Zhang W. Cheng J. Yang X. Zhu K. Wang Y. (2017). A *Pseudomonas* T6SS effector recruits PQS-containing outer membrane vesicles for iron acquisition. *Nat. Commun.* 8:14888. 10.1038/ncomms14888 28348410 PMC5379069

[B31] Lin J. S. Ma L. S. Lai E. M. (2013). Systematic dissection of the *Agrobacterium* type VI secretion system reveals machinery and secreted components for subcomplex formation. *PLoS One* 8:e67647. 10.1371/journal.pone.0067647 23861778 PMC3702570

[B32] Lin J. S. Wu H. H. Hsu P. H. Ma L. S. Pang Y. Y. Tsai M. D. (2014). Fha interaction with phosphothreonine of TssL activates type VI secretion in *Agrobacterium tumefaciens*. *PLoS Pathog.* 10:e1003991. 10.1371/journal.ppat.1003991 24626341 PMC3953482

[B33] Lossi N. S. Manoli E. Forster A. Dajani R. Pape T. Freemont P. (2013). The HsiB1C1 (TssB-TssC) complex of the *Pseudomonas aeruginosa* type VI secretion system forms a bacteriophage tail sheathlike structure. *J. Biol. Chem.* 288 7536–7548. 10.1074/jbc.M112.439273 23341461 PMC3597794

[B34] Ma J. Sun M. Pan Z. Lu C. Yao H. (2018). Diverse toxic effectors are harbored by *vgrG* islands for interbacterial antagonism in type VI secretion system. *Biochim. Biophys. Acta Gen. Subj.* 1862 1635–1643. 10.1016/j.bbagen.2018.04.010 29674124

[B35] Ma L. S. Hachani A. Lin J. S. Filloux A. Lai E. M. (2014). *Agrobacterium tumefaciens* deploys a superfamily of type VI secretion DNase effectors as weapons for interbacterial competition in planta. *Cell Host Microbe* 16 94–104. 10.1016/j.chom.2014.06.002 24981331 PMC4096383

[B36] Marchi M. Boutin M. Gazengel K. Rispe C. Gauthier J. P. Guillerm-Erckelboudt A. Y. (2013). Genomic analysis of the biocontrol strain *Pseudomonas fluorescens* Pf29Arp with evidence of T3SS and T6SS gene expression on plant roots. *Environ. Microbiol. Rep.* 5 393–403. 10.1111/1758-2229.12048 23754720

[B37] McCarter S. M. Jones J. B. Gitaitis R. D. Smitley D. R. (1983). Survival of *Pseudomonas syringae* pv. tomato in association with tomato seed, soil, host tissue, and epiphytic weed hosts in Georgia. *Phytopathology* 73 1393–1398.

[B38] Michiels J. Xi C. Verhaert J. Vanderleyden J. (2002). The functions of Ca^2+^ in bacteria: a role for EF-hand proteins? *Trends Microbiol.* 10 87–93. 10.1016/s0966-842x(01)02284-3 11827810

[B39] Miyata S. T. Kitaoka M. Brooks T. M. Mcauley S. B. Pukatzki S. (2011). Vibrio cholerae requires the type VI secretion system virulence factor VasX to kill *Dictyostelium discoideum*. *Infect. Immun.* 79 2941–2949. 10.1128/IAI.01266-10 21555399 PMC3191968

[B40] Mougous J. D. Cuff M. E. Raunser S. Shen A. Zhou M. Gifford C. A. (2006). A virulence locus of *Pseudomonas aeruginosa* encodes a protein secretion apparatus. *Science* 312 1526–1530. 10.1126/science.1128393 16763151 PMC2800167

[B41] Mougous J. D. Gifford C. A. Ramsdell T. L. Mekalanos J. J. (2007). Threonine phosphorylation post-translationally regulates protein secretion in *Pseudomonas aeruginosa*. *Nat. Cell Biol.* 9 797–803. 10.1038/ncb1605 17558395

[B42] Pukatzki S. Ma A. T. Sturtevant D. Krastins B. Sarracino D. Nelson W. C. (2006). Identification of a conserved bacterial protein secretion system in *Vibrio cholerae* using the *Dictyostelium* host model system. *Proc. Natl. Acad. Sci. U.S.A.* 103 1528–1533. 10.1073/pnas.0510322103 16432199 PMC1345711

[B43] Rico A. Preston G. M. (2008). *Pseudomonas syringae* pv. tomato DC3000 uses constitutive and apoplast-induced nutrient assimilation pathways to catabolize nutrients that are abundant in the tomato apoplast. *Mol. Plant Microbe Interact.* 21 269–282. 10.1094/MPMI-21-2-0269 18184070

[B44] Russell A. B. Peterson S. B. Mougous J. D. (2014). Type VI secretion system effectors: poisons with a purpose. *Nat. Rev. Microbiol.* 12 137–148. 10.1038/nrmicro3185 24384601 PMC4256078

[B45] Russell A. B. Singh P. Brittnacher M. Bui N. K. Hood R. D. Carl M. A. (2012). A widespread bacterial type VI secretion effector superfamily identified using a heuristic approach. *Cell Host Microbe* 11 538–549. 10.1016/j.chom.2012.04.007 22607806 PMC3358704

[B46] Sabala I. Jagielska E. Bardelang P. T. Czapinska H. Dahms S. O. Sharpe J. A. (2014). Crystal structure of the antimicrobial peptidase lysostaphin from *Staphylococcus simulans*. *FEBS J.* 281 4112–4122. 10.1111/febs.12929 25039253 PMC4286107

[B47] Salomon D. Klimko J. A. Trudgian D. C. Kinch L. N. Grishin N. V. Mirzaei H. (2015). Type VI secretion system toxins horizontally shared between marine bacteria. *PLoS Pathog.* 11:e1005128. 10.1371/journal.ppat.1005128 26305100 PMC4549250

[B48] Sana T. G. Soscia C. Tonglet C. M. Garvis S. Bleves S. (2013). Divergent control of two type VI secretion systems by RpoN in *Pseudomonas aeruginosa*. *PLoS One* 8:e76030. 10.1371/journal.pone.0076030 24204589 PMC3804575

[B49] Sarris P. F. Skandalis N. Kokkinidis M. Panopoulos N. J. (2010). *In silico* analysis reveals multiple putative type VI secretion systems and effector proteins in *Pseudomonas syringae* pathovars. *Mol. Plant Pathol.* 11 795–804. 10.1111/j.1364-3703.2010.00644.x 21091602 PMC6640432

[B50] Schafer A. Tauch A. Jager W. Kalinowski J. Thierbach G. Puhler A. (1994). Small mobilizable multi-purpose cloning vectors derived from the *Escherichia coli* plasmids pK18 and pK19: selection of defined deletions in the chromosome of *Corynebacterium glutamicum*. *Gene* 145 69–73. 10.1016/0378-1119(94)90324-7 8045426

[B51] Shalom G. Shaw J. G. Thomas M. S. (2007). *In vivo* expression technology identifies a type VI secretion system locus in *Burkholderia pseudomallei* that is induced upon invasion of macrophages. *Microbiology* 153, 2689–2699. 10.1099/mic.0.2007/006585-0 17660433

[B52] Shneider M. M. Buth S. A. Ho B. T. Basler M. Mekalanos J. J. Leiman P. G. (2013). PAAR-repeat proteins sharpen and diversify the type VI secretion system spike. *Nature* 500 350–353. 10.1038/nature12453 23925114 PMC3792578

[B53] Si M. Wang Y. Zhang B. Zhao C. Kang Y. Bai H. (2017). The type VI secretion system engages a redox-regulated dual-functional heme transporter for zinc acquisition. *Cell Rep.* 20 949–959. 10.1016/j.celrep.2017.06.081 28746878

[B54] Silverman J. M. Agnello D. M. Zheng H. Andrews B. T. Li M. Catalano C. E. (2013). Haemolysin coregulated protein is an exported receptor and chaperone of type VI secretion substrates. *Mol. Cell.* 51 584–593. 10.1016/j.molcel.2013.07.025 23954347 PMC3844553

[B55] Suarez G. Sierra J. C. Sha J. Wang S. Erova T. E. Fadl A. A. (2008). Molecular characterization of a functional type VI secretion system from a clinical isolate of *Aeromonas hydrophila*. *Microb. Pathog.* 44 344–361. 10.1016/j.micpath.2007.10.005 18037263 PMC2430056

[B56] Thomas J. Watve S. S. Ratcliff W. C. Hammer B. K. (2017). Horizontal gene transfer of functional type VI killing genes by natural transformation. *mBio* 8:e00654-17. 10.1128/mBio.00654-17 28743812 PMC5527308

[B57] Trunk K. Peltier J. Liu Y. C. Dill B. D. Walker L. Gow N. A. R. (2018). The type VI secretion system deploys antifungal effectors against microbial competitors. *Nat. Microbiol.* 3 920–931. 10.1038/s41564-018-0191-x 30038307 PMC6071859

[B58] Unterweger D. Kitaoka M. Miyata S. T. Bachmann V. Brooks T. M. Moloney J. (2012). Constitutive type VI secretion system expression gives *Vibrio cholerae* intra- and interspecific competitive advantages. *PLoS One* 7:e48320. 10.1371/journal.pone.0048320 23110230 PMC3482179

[B59] Unterweger D. Kostiuk B. Otjengerdes R. Wilton A. Diaz-Satizabal L. Pukatzki S. (2015). Chimeric adaptor proteins translocate diverse type VI secretion system effectors in *Vibrio cholerae*. *EMBO J.* 34 2198–2210. 10.15252/embj.201591163 26194724 PMC4557670

[B60] Wang T. Si M. Song Y. Zhu W. Gao F. Wang Y. (2015). Type VI secretion system transports Zn2+ to combat multiple stresses and host Immunity. *PLoS Pathog.* 11:e1005020. 10.1371/journal.ppat.1005020 26134274 PMC4489752

[B61] Whitney J. C. Beck C. M. Goo Y. A. Russell A. B. Harding B. N. De Leon J. A. (2014). Genetically distinct pathways guide effector export through the type VI secretion system. *Mol. Microbiol.* 92 529–542. 10.1111/mmi.12571 24589350 PMC4049467

[B62] Winsor G. L. Griffiths E. J. Lo R. Dhillon B. K. Shay J. A. Brinkman F. S. (2016). Enhanced annotations and features for comparing thousands of *Pseudomonas* genomes in the *Pseudomonas genome database*. *Nucleic Acids Res.* 44 D646–D653. 10.1093/nar/gkv1227 26578582 PMC4702867

[B63] Xin X. F. He S. Y. (2013). *Pseudomonas syringae* pv. tomato DC3000: a model pathogen for probing disease susceptibility and hormone signaling in plants. *Annu. Rev. Phytopathol.* 51 473–498. 10.1146/annurev-phyto-082712-102321 23725467

[B64] Yanez M. Gil-Longo J. Campos-Toimil M. (2012). Calcium binding proteins. *Adv. Exp. Med. Biol.* 740 461–482. 10.1007/978-94-007-2888-2_19 22453954

[B65] Yang W. Wang L. Zhang L. Qu J. Wang Q. Zhang Y. (2015). An invasive and low virulent *Edwardsiella tarda esrB* mutant promising as live attenuated vaccine in aquaculture. *Appl. Microbiol. Biotechnol.* 99 1765–1777. 10.1007/s00253-014-6214-5 25431010

[B66] Zepeda-Rivera M. A. Saak C. C. Gibbs K. A. (2018). A proposed chaperone of the bacterial type VI secretion system functions to constrain a self-identity protein. *J. Bacteriol.* 200:e00688-17. 10.1128/JB.00688-17 29555703 PMC6018363

[B67] Zheng J. Ho B. Mekalanos J. J. (2011). Genetic analysis of anti-amoebae and anti-bacterial activities of the type VI secretion system in *Vibrio cholerae*. *PLoS One* 6:e23876. 10.1371/journal.pone.0023876 21909372 PMC3166118

[B68] Zheng J. Leung K. Y. (2007). Dissection of a type VI secretion system in *Edwardsiella tarda*. *Mol. Microbiol.* 66 1192–1206. 10.1111/j.1365-2958.2007.05993.x 17986187

[B69] Zoued A. Durand E. Brunet Y. R. Spinelli S. Douzi B. Guzzo M. (2016). Priming and polymerization of a bacterial contractile tail structure. *Nature* 531 59–63. 10.1038/nature17182 26909579

