# Supplementary material for: HSI-II Gene Cluster of Pseudomonas syringae pv. tomato DC3000 Encodes a Functional Type VI Secretion System Required for Interbacterial Competition
Source: Front Microbiol. 2020 Jun 3;11:1118. doi: 10.3389/fmicb.2020.01118 (PMC7283901; doi:10.3389/fmicb.2020.01118)
Supplement: Supplementary file 1 [file Data_Sheet_1.PDF]

TABLE S1. Bacterial strains and plasmids used in this study.

Designation	Relevant characteristics	Source and/or reference
**Strains**		
Agrobacterium tumefaciens C58		E. M. Lai, Taiwan
Dickeya dadantii CAS9		W.L. Deng, Taiwan
Escherichia coli		
K-12 MG1655	F^-, λ^-, *ilvG*$^-$, *rfb-50*, *rph-1*	
DH5α	F^-, φ80dl*lacZ* Δ M15, Δ (*lacZYA* - *argF*)*U169*, *recA1*, *endA1*, *hsdR17* (rK$^-$, mK$^+$), *phoA*, *supE44*, λ^-, *thi-1*, *gyrA96*, *relA1*	Stratagen, Germany
S17-1	*recA, thi, pro, hsdR* M^+ RP4: 2-Tc:Mu:Km-Tn7 λpir	Laboratory stock
Pseudomonas putida NLA7		Laboratory stock
Pseudomonas syringe pv. *tomato* (*Pst*) DC3000		
Wild type	Rifampicin-resistant derivative	Cupple, 1986
ΔHSI-I	In frame-deletion of PSPTO_2542 to 2554, Rif^R	This study
ΔHSI-II	In frame-deletion of PSPTO_5414 to 5427, Rif^R	This study
ΔHSI-I/II	In frame-deletion of PSPTO_2542 to 2554 and 5414 to 5427, Rif^R	This study
Δ5413	In frame-deletion of PSPTO_5413, Rif^R	This study
Δ5414	In frame-deletion of PSPTO_5414, Rif^R	This study
Δ5415	In frame-deletion of PSPTO_5415, Rif^R	This study
Δ5416	In frame-deletion of PSPTO_5416, Rif^R	This study
Δ5417	In frame-deletion of PSPTO_5417, Rif^R	This study
Δ5418	In frame-deletion of PSPTO_5418, Rif^R	Haapalainen et al., 2012
Δ5419	In frame-deletion of PSPTO_5419, Rif^R	This study
Δ5420	In frame-deletion of PSPTO_5420, Rif^R	This study
Δ5421	In frame-deletion of PSPTO_5421, Rif^R	This study
Δ5422	In frame-deletion of PSPTO_5422, Rif^R	This study
Δ5423	In frame-deletion of PSPTO_5423, Rif^R	This study
Δ5424	In frame-deletion of PSPTO_5424, Rif^R	This study
Δ5425	In frame-deletion of PSPTO_5425, Rif^R	This study
Δ5426	In frame-deletion of PSPTO_5426, Rif^R	This study

TABLE S1. Bacterial strains and plasmids used in this study. (Continued)

Designation	Relevant characteristics	Source and/or reference
**Strains**		
Δ5427	In frame-deletion of PSPTO_5427, Rif^R	This study
Δ5645	In frame-deletion of PSPTO_5645, Rif^R	This study
Δ5430	In frame-deletion of PSPTO_5430, Rif^R	This study
Δ5431	In frame-deletion of PSPTO_5431, Rif^R	This study
Δ5432	In frame-deletion of PSPTO_5432, Rif^R	This study
Δ5433	In frame-deletion of PSPTO_5433, Rif^R	This study
Δ5434	In frame-deletion of PSPTO_5434, Rif^R	This study
Δ5435	In frame-deletion of PSPTO_5435, Rif^R	Haapalainen et al., 2012
Δ5436	In frame-deletion of PSPTO_5436, Rif^R	This study
Δ5437	In frame-deletion of PSPTO_5437, Rif^R	This study
Δ5438	In frame-deletion of PSPTO_5438, Rif^R	This study
Δ5439	In frame-deletion of PSPTO_5439, Rif^R	This study
Δ5645/5646	In frame-deletion of PSPTO_5645 and 5646, Rif^R	This study
P. s. pv. *tomato* T1	Wild type, Rif^R	Laboratory stock
P. s. pv. *syringae* 61	Wild type, Rif^R	Laboratory stock
P. savastanoi pv. *phaseolicola* 1448a	Wild type, Rif^R	Laboratory stock
P. viridiflava	Isolated from tomato leaves	W. L. Deng, Taiwan
Xanthomonas euvescatoria XvT147	Isolated from diseased tomato plants	W. L. Deng, Taiwan
Xanthomonas oryzae pv. *oryzae* 20	Isolated from diseased rice plants	W. L. Deng, Taiwan
**Plasmids**		
pK18*mobsac*	Suicide vector, Km^R	Schafer et al. 1994
pCPP45	Broad-host-range vector, Tc^R	
pCHUB78	Vector containing promoterless *uidA* gene, Gm^R	W. L. Deng, Taiwan
pGFP-TIR	*gfp* gene under the control of a *lac* promoter, Gm^R	W. L. Deng, Taiwan

Rif^R, rifampicin resistant; Km^R, kanamycin resistant; Gm^R, gentamicin resistant; Tc^R, tetracycline resistant.

TABLE S2. Primers used for mutant construction.

Primers	Sequence (5'→3')[1]	Feature
Pto_5413mt-1	CCGGATCCCACCTAATAGCCCCGGTG	BamHI site
Pto_5413mt-2	*TTCGACATACTTATAACTC*CAGATGCATATACAAGCT	
Pto_5413mt-3	GAGTTATAAGTATGTCGAA	
Pto_5413mt-4	CCTCTAGACACCCCCTTGAGCCAAAT	XbaI site
Pto_5414mt-1	CCTCTAGATCGAAAACGACCAGAAAATC	XbaI site
Pto_5414mt-2	*TCCCGCAAATTAATAGCA*CGCGAATACGCTATTTTTTT	
Pto_5414mt-3	TGCTATTAATTTGCGGGATG	
Pto_5414mt-4	CCTCTAGATTCCTCCGCATCAGTGAACT	XbaI site
Pto_5415mt-1	CCGGATCCACCGAATACCTGACCGGACG	BamHI site
Pto_5415mt-2	*TTTTTTTTGAAAACACGATTCAT*TGTTTGCTCCCTGTTCGGTT	
Pto_5415mt-3	ATGAATCGTGTTTTCAAAAAAAA	
Pto_5415mt-4	CCTCTAGAATATGAATACCACCGTGCCA	XbaI site
Pto_5416mt-1	CCGGATCCATTCGTCAATGTCTGCACTGG	BamHI site
Pto_5416mt-2	*GTTCGGTTGTCATCCGTT*GTTGCTGTCCTGCTCTTCGT	
Pto_5416mt-3	AACGGATGACAACCGAACAG	
Pto_5416mt-4	CCTCTAGATGGAAGTGGTAGTGAACCCC	XbaI site
Pto_5417mt-1	CCGGATCCTAACTACATGCGTCCGTTCG	BamHI site
Pto_5417mt-2	*GCACGAGCTCAGTCATTT*TACCAGCATCAAAGCTGCCC	
Pto_5417mt-3	AAATGACTGAGCTCGTGCGC	
Pto_5417mt-4	CCTCTAGACATGTGAATAAGCCAGTGCG	XbaI site
Pto_5418mt-1	CCTCTAGAACACCCATGAACCGCTCGCC	XbaI site
Pto_5418mt-2	*CAAGGGCTGGCTACCAGCAT*TTACGGCTGTGACTGCGGCTGGAC	
Pto_5418mt-3	ATGCTGGTAGCCAGCCCTTGGCGC	
Pto_5418mt-4	CCAAGCTTGCTGCGCCGACATCTGCTGA	HindIII site
Pto_5419mt-1	CCGGATCCAAGAGTTGTATCGCACGCTG	BamHI site
Pto_5419mt-2	*CCCTGTTTACGGCTGTGA*GCTCATGTCGGTCAGTTCCT	
Pto_5419mt-3	TCACAGCCGTAAACAGGGAC	
Pto_5419mt-4	CCTCTAGAATCGGCGAAGTACCAGTCAC	XbaI site
Pto_5420mt-1	CCGGATCCTCCACACTCCACACTGACCA	BamHI site
Pto_5420mt-2	*TGCTCATGTCGGTCAGTT*TTTCATGGTCAGTCATCTGC	
Pto_5420mt-3	AACTGACCGACATGAGCAAG	
Pto_5420mt-4	CCTCTAGATACATCAATTCCAGCATCGG	XbaI site
Pto_5421mt-1	CCGGATCCTAAAGAAGCGTTGGCTTTGA	BamHI site
Pto_5421mt-2	*TCATGGTCAGTCATCTGC*AGACATGCGCATCATCCTTG	

TABLE S2. Primers used for mutant construction. (Continued)

Primers	Sequence (5'→3')	Feature
Pto_5421mt-3	GCAGATGACTGACCATGAAATC	
Pto_5421mt-4	CCTCTAGATGTCGAGTACTTCGCAAACC	XbaI site
Pto_5422mt-1	CCGGATCCTATACGTTTCCCGGCAATGT	BamHI site
Pto_5422mt-2	*ACATGCGCATCATCCTTG*TTCCATGATGATGCTCCTTG	
Pto_5422mt-3	CAAGGATGATGCGCATGTCT	
Pto_5422mt-4	CCTCTAGACCTTCCTGCCAAATGACTTT	XbaI site
Pto_5423mt-1	CCGAATTCACCGTCTTGCGCAATTTCC	EcoRI site
Pto_5423mt-2	*TGATGCTCCTTGCGGTCA*AAAGCGCCACCCCTTTCG	
Pto_5423mt-3	TGACCGCAAGGAGCATCA	
Pto_5423mt-4	CCTCTAGAACGCTGCTTGCCATCATC	XbaI site
Pto_5424mt-1	CCTCTAGACCGAGTTTCAGGAGAAGCA	XbaI site
Pto_5424mt-2	*ATTGCTTTTGCCTAACCATT*CTGCGGCATCTGAGTGAA	
Pto_5424mt-3	AATGGTTAGGCAAAAGCAAT	
Pto_5424mt-4	CCAAGCTTTGCCATCATCCAAGGAGA	HindIII site
Pto_5425mt-1	CCGGATCCGATCTGGAAGAAATATCGC	BamHI site
Pto_5425mt-2	*ACATTCACCTCAGGCAAA*GCTCATCAGTCATTGATC	
Pto_5425mt-3	TTTGCCTGAGGTGAATGT	
Pto_5425mt-4	CCTCTAGACAACACAGTGTACGGGCT	XbaI site
Pto_5426mt-1	CCGGATCCAAACCCTGTCGATCGAGTTG	BamHI site
Pto_5426mt-2	*AGTCATTGATCCTTAATG*CGTGGTGTCCATTGGTACAC	
Pto_5426mt-3	CATTAAGGATCAATGACTGATGAG	
Pto_5426mt-4	CCTCTAGATGAGTGAAACGCTCCAACAG	XbaI site
Pto_5427mt-1	CCGAATTCCTGAGTGGCTGCTTTGGC	EcoRI site
Pto_5427mt-2	*GAGGCCCATACGTGGTGT*ACTGAACGCAGGCAACGG	
Pto_5427mt-3	ACACCACGTATGGGCCTCC	
Pto_5427mt-4	CCTCTAGATGAGCAAGCCCAGGTAGG	XbaI site
Pto_5430mt-1	CCGAATTCTCAGCCTGAACGTGCGC	EcoRI site
Pto_5430mt-2	*TCATAATCAACCGATCAC*TGCTGCGGGTTTTCCA	
Pto_5430mt-3	GTGATCGGTTGATTATGATTCTAA	
Pto_5430mt-4	CCTCTAGACTCATCCAGCTCACCCCC	XbaI site
Pto_5431mt-1	CCGAATTCATCCCTACCGAGGTTCTGGT	EcoRI site
Pto_5431mt-2	*TCCTTAATTAACCTTCAC*TCCAGTCATGAGTGAGCTCC	
Pto_5431mt-3	GTGAAGGTTAATTAAGGAGATTCG	
Pto_5431mt-4	CCTCTAGACTGACTGAGGCTCGATGATAA	XbaI site

TABLE S2. Primers used for mutant construction. (Continued)

Primers	Sequence (5'→3')	Feature
Pto_5432mt-1	CCGAATTCCGCATCAACGTCACTTTTAAA	EcoRI site
Pto_5432mt-2	*CTCCGTTTACTCTTTGTC*GCTTGGCATGAATTTGACTT	
Pto_5432mt-3	GACAAAGAGTAAACGGAGCTCA	
Pto_5432mt-4	CCTCTAGAGTTGCTGGAGCCAGCAAC	XbaI site
Pto_5433mt-1	CCGAATTCCATCATCGAATTGCGCTATC	EcoRI site
Pto_5433mt-2	*GCTGATAGGGACTCAAGC*GGCCATGGCTTTTCTCCT	
Pto_5433mt-3	GCTTGAGTCCCTATCAGCCA	
Pto_5433mt-4	CCTCTAGAGGCGAGCTTGGAGACATG	XbaI site
Pto_5434mt-1	CCGAATTCGGCGATATATCCACAGGTTACTAA	EcoRI site
Pto_5434mt-2	*TGGCCCTAAGGCCTATTC*GGACATCCATGACCTTCG	
Pto_5434mt-3	GAATAGGCCTTAGGGCCAC	
Pto_5434mt-4	CCTCTAGAGCTTTCAGGCCCAGTTCA	XbaI site
Pto_5435mt-1	CCGGATCCTTCCAGCTCGACCAAGAGATC	BamHI site
Pto_5435mt-2	*TGATTGGCCGGCGCGAACAT*GGAGGTGCTCCTTGCTGGAT	
Pto_5435mt-3	ATGTTCGCGCCGGCCAATCA	
Pto_5435mt-4	CCTCTAGATCAGATCGCACACGCACCGT	XbaI site
Pto_5436mt-1	CCGGATCCTCACGAAGACCAAGTGATGGTT	BamHI site
Pto_5436mt-2	*AGGCCTGCGGAGtCAAAAGA*CCCTTGTTCCTCGTGCAGTG	
Pto_5436mt-3	TCTTTTGACTCCGCAGGCCT	
Pto_5436mt-4	CCTCTAGAGCCAGTCTTTCACCACTGGC	XbaI site
Pto_5437mt-1	CCGGATCCAACGAACGACACGACACAG	BamHI site
Pto_5437mt-2	*CAACGCGATCCATCAGCC*GAGATGCTCACCCACCTG	
Pto_5437mt-3	GGCTGATGGATCGCGTTG	
Pto_5437mt-4	CCTCTAGAAAGCGCTTTGATTGGACC	XbaI site
Pto_5438mt-1	CCGGATCCTACGATACCTGGCAGCCG	BamHI site
Pto_5438mt-2	*TATTCATTAATTTTAGGT*ATCCATCAGCCTTGCTCC	
Pto_5438mt-3	ACCTAAAATTAATGAATAGGGCA	
Pto_5438mt-4	CCTCTAGAGCGATACCGGTCGGTG	XbaI site
Pto_5439mt-1	CCGAATTCGGCATAAAATGTTTTCAGACG	EcoRI site
Pto_5439mt-2	*ATCGCCCACAGATTTTGT*ATACCGGTCGGTGGTTTT	
Pto_5439mt-3	ACAAAATCTGTGGGCGAT	
Pto_5439mt-4	CCTCTAGATCTCGATGAGAAAGCGCT	XbaI site
Pto_5645mt-1	CCGAATTCAGGAGATTCGTATGTCTGGAAA	EcoRI site
Pto_5645mt-2	*ACATTATCCCCTCACAAA*TGTCATAAATGTGCTCGCTATC	
Pto_5645mt-3	TTTGTGAGGGGATAATGTTTTC	
Pto_5645mt-4	CCTCTAGACAAATCAGGAAACCTTTTTCAG	XbaI site

TABLE S2. Primers used for mutant construction. (Continued)

Primers	Sequence (5’→3’)	Feature
Pto_5646mt-1	CC<u>GAATTC</u>CGTTAGATTTAATGGTAAGGGG	EcoRI site
Pto_5646mt-2	*TTCACTGTTCCGCTACTC*AGAAAACATTATCCCCTCACAA	
Pto_5646mt-3	GAGTAGCGGAACAGTGAATCG	
Pto_5646mt-4	CC<u>TCTAGA</u>ATAGCCATTGATGACCTGCA	XbaI site

[1]Sequences underlined are sites recognized by restriction endonucleases indicated. Characters of each Pto_XXXXmt-2 primer in italic are sequences complementary to corresponding Pto_XXXXmt-3 primer (“XXXX” indicates the gene ID).

Table S3. Primers used for semi-quantitative RT-PCR to determine the extent of HSI-II gene cluster.

Primers	Sequence (5'→3')	Feature[1]
Pto_HSI-II-i-1F	CTGCTGGCGATGGGAG	To amplify the intergenic region 'e'
Pto_HSI-II-i-1R	ATCTCCAGGCGCAGGC	(1724 bp)
Pto_HSI-II-i-2F	GGGTGACTTAAATGGTTAGGC	To amplify the intergenic region 'd'
Pto_HSI-II-i-2R	CGAATTGCTTGGTGTTCA	(304 bp)
Pto_HSI-II-i-3F	AATGCACCGCCTGAGAAA	To amplify the intergenic region 'c'
Pto_HSI-II-i-3R	GCATACGCACGGTCAAGA	(145 bp)
Pto_HSI-II-i-4F	CGACGTTTCAACAGCTGG	To amplify the intergenic region 'b'
Pto_HSI-II-i-4R	AGGTGTTTCTGCAGGTGA	(992 bp)
Pto_HSI-II-i-5F	CGATACAATCACTTGAAGGC	To amplify the intergenic region 'a'
Pto_HSI-II-i-5R	TTTCTCGACTTTGGCGTC	(1950 bp)
Pto_HSI-II-ii-2F	CATCATCGAATTGCGCTATC	To amplify the region 'h' (1584 bp)
Pto_HSI-II-ii-2R	GGCGAGCTTGGAGACATG	
Pto_HSI-II-ii-3F	GTGAAGGTTAATTAAGGAGATTCG	To amplify the region 'g' (611 bp)
Pto_HSI-II-ii-3R	CAAACTGGAAGCTACTGT	
Pto_HSI-II-ii-4F	AGCAATACTCCATATGAAGTGA	To amplify the intergenic region 'f'
Pto_HSI-II-ii-4R	CGAAGGTCATCTGTTTCA	(224 bp)
Pto_HSI-II-iii-1F	TTCGGGTTCCGATGACTG	To amplify the intergenic region 'i'
Pto_HSI-II-iii-1R	GTGAGTCTGATTGGCCGG	(198 bp)
Pto_HSI-II-iii-2F	GAGAAGTGCAAGGACGGT	To amplify the intergenic region 'j'
Pto_HSI-II-iii-2R	CCGCACTCACAACCAGAT	(110 bp)
Pto_HSI-II-iii-3F	GCCGAAGATATAATGCCA	To amplify the intergenic region 'k'
Pto_HSI-II-iii-3R	GGGTACCCAAATGGTCAA	(757 bp)

[1]The region amplified by each primer pair is in Figure 3A.

Table S4. Summary of the effect of each mutant in the HSI-II gene cluster

strain	Hcp2 accumulation[1]	Hcp2 secretion[2]	Competition assay[3]	
			E. coli MG1655	*Psph* 1448a
Wild type	+	+	++	++
ΔHSI-II	-	-	-	-
Δ5413	+	+	-	+
Δ5414	+	+	++	++
Δ5415	+	+	++	+
Δ5416	+	-	-	+
Δ5417	+	+	++	++
Δ5418	+	-	-	-
Δ5419	+	-	-	-
Δ5420	+	-	-	-
Δ5421	+	(+)	-	-
Δ5422	+	-	-	-
Δ5423	+	-	-	-
Δ5424	-	-	-	-
Δ5425	+	-	-	-
Δ5426	+	+	++	++
Δ5427	(+)	-	-	-
Δ5645	+	+	++	++
Δ5430	+	+	++	++
Δ5431	+	-	-	-
Δ5432	+	-	-	-
Δ5433	+	-	-	-
Δ5434	+	-	-	-
Δ5435	-	-	-	-
Δ5436	+	(+)	-	-
Δ5437	+	+	++	++
Δ5438	+	+	++	++

[1]Based on the results presented in "Cell pellet" of Figure 4. +, Hcp2 accumulated; (+), reduced Hcp2 amount; -, no Hcp2 accumulation.

[2]Based on the results presented in "Secreted proteins" of Figure 4. +, Hcp2 secreted; (+) reduced Hcp2 amount; -, no Hcp2 secretion.

[3]Based on the bacterial growth in the competition assays. For assays using *E. coli* K-12 MG1655 and *P. savastanoi* pv. *phaseolicola* 1448a as competitors (Figure 5): ++, strong reduction; +, reduction; -, weak/no reduction of competitor growth (compared to no co-inoculation control, "*E. coli* only" or "*Pph* only"), ++, strong reduction; +, reduction; -, weak/no reduction of *Pst*DC3000 growth (compared to no co-inoculation control, "*E. coli* only" or "*Psph* only").

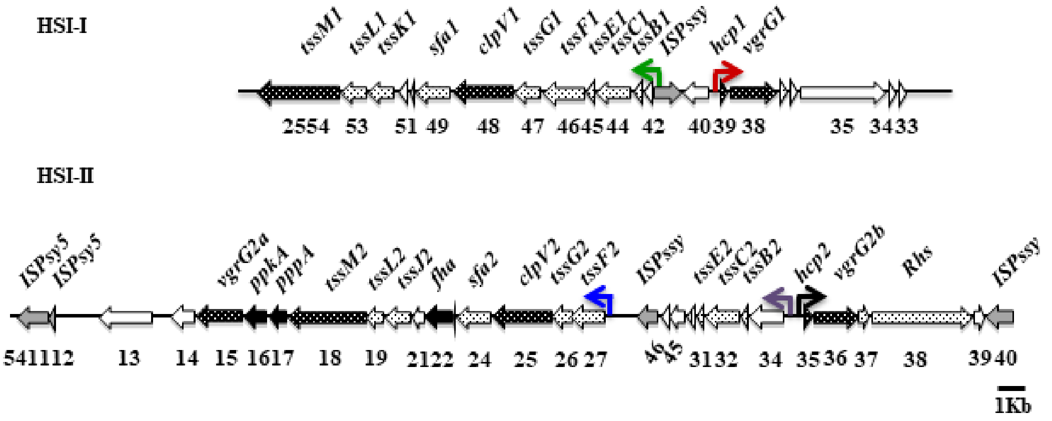

Figure S1. The predicted promoters in the HSI-I and HSI-II gene clusters. Schematic diagram of 22 (*PSPTO_2533 to PSPTO_2554)* and 27 genes (*PSPTO_5413* to *PSPTO_5439*) of the HSI-I and HSI-II gene clusters, respectively. Black arrows are open reading frames (ORFs) orthologous to the core T6SS components (names are given above and gene ID numbers are below) and genes coding for putative transposases are shaded gray. Dotted arrows represent ORFs found in both HSI-I and HSI-II gene clusters. Arrows with diagonal lines represent ORFs orthologous to the regulatory T6SS components, including PpkA, PppA and Fha. Colored arrows designate five predicted promoters, P*HSI-I* (green), P*hcp1*(red), P*HSI-I-i* (blue), P*HSI-II-ii* (grey) and P*HSI-II-iii* (black), and the direction of transcription.

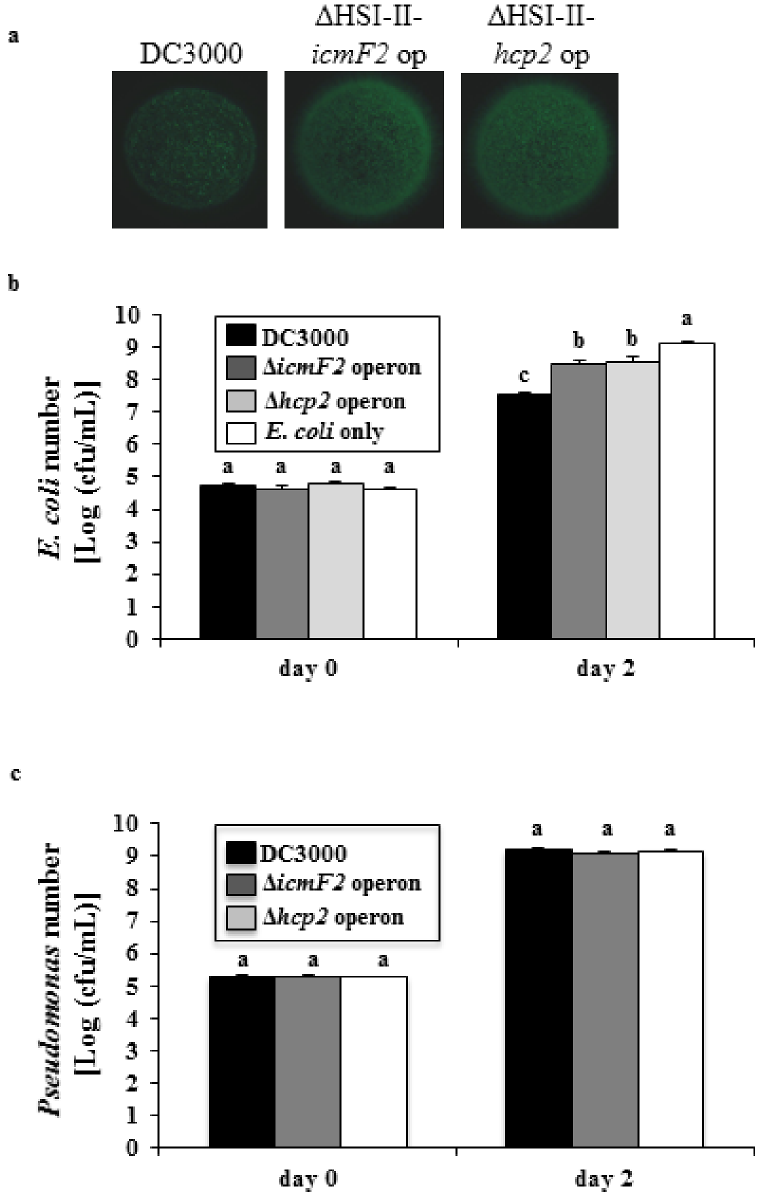

Figure S2. Deletion of the first and third operons in the HSI-II gene cluster decreases growth fitness on co-incubation with *E. coli* MG1655. The competition ability of operon mutants ΔHSI-II-i and ΔHSI-II-iii was assessed on co-incubation with *E. coli* MG1655 expressing GFP in a ratio of 10:1. (a) The GFP signal was captured two days after co-incubation on King's media B. Bacterial numbers of *E. coli* MG1655 (expressing GFP) (b) and each *Pst* DC3000 strain (c) was measured on day 0 and day 2 during co-incubation. Data are mean ± SD and were analyzed by one-way ANOVA followed by Tukey's HSD test. Bars with different letters above indicate significant difference at $p<0.05$. This experiment was performed three times with similar results.

a

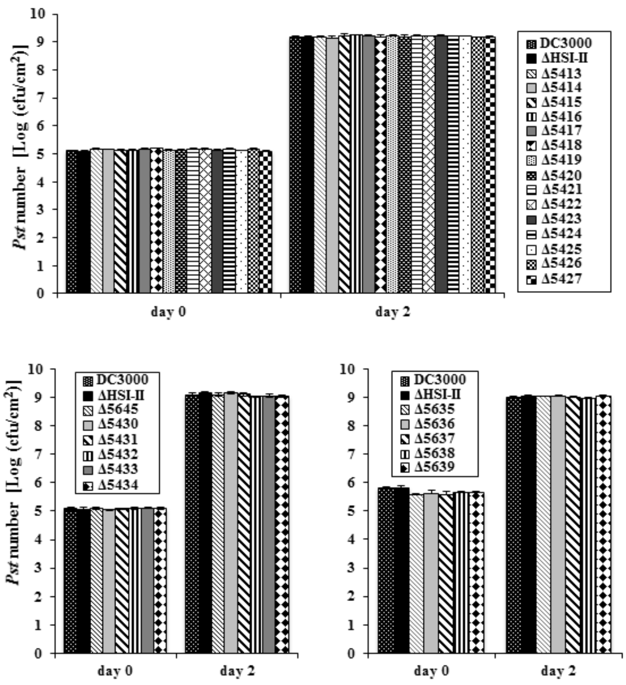

b

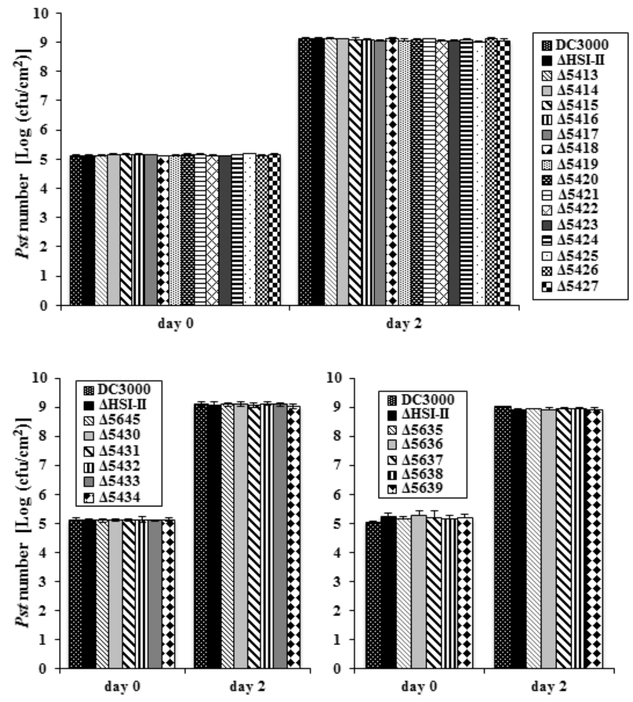

Fig. S3. Deletion of each gene in the HSI-II gene cluster did not affect the bacterial number of each *Pst*DC3000 strain in the interbacterial competition assay. After co-incubation with GFP-expressing *E. coli* MG1655 (a) or *Psph* 1448a (b) for 2 days, the bacterial number of each *Pst* DC3000 strain was measured. Data are mean± SD (n=3), and analyzed by ANOVA test. This experiment was conducted three times with similar results.
